# Rising flood risks in semiarid South Asia driven by changing intraseasonal oscillations under global warming

**DOI:** 10.1126/sciadv.aea0082

**Published:** 2026-04-03

**Authors:** Jinhui Xie, Pang-Chi Hsu, June-Yi Lee, Lu Wang, Pallav Ray, Lei Zhou, Baosheng Li

**Affiliations:** ^1^ State Key Laboratory of Climate System Prediction and Risk Management/Key Laboratory of Meteorological Disaster, Ministry of Education/Collaborative Innovation Center on Forecast and Evaluation of Meteorological Disasters, Nanjing University of Information Science and Technology, Nanjing, Jiangsu, China.; ^2^ Center for Climate Physics, Institute for Basic Science, Busan, South Korea.; ^3^ Research Center for Climate Sciences, Pusan National University, Busan, South Korea.; ^4^ Florida Institute of Technology, Melbourne, FL, USA.; ^5^ School of Oceanography, Shanghai Jiao Tong University, Shanghai, China.; ^6^ School of Marine Sciences, Sun Yat-sen University, Zhuhai, Guangdong, China.

## Abstract

Northwestern South Asia, encompassing the historically semiarid regions of Pakistan and northwest India, has seen increased persistent heavy rainfall and catastrophic flooding in recent decades. While most studies emphasize seasonal mean changes, the role of subseasonal dynamics remains unclear. Here, we present observational evidence that the northward-propagating monsoon intraseasonal oscillation (ISO) has strengthened and penetrated farther inland, with its convective anomalies amplifying rainfall extremes. Simultaneously, the southeastward-propagating mid-latitude ISO along the westerly jet has slowed, prolonging anomalous circulations that sustain rainfall episodes. Together, these ISOs account for ~44% of the observed increase in flood frequency, a contribution comparable to that from mean-state changes (~40%). CMIP6 projections suggest that these ISO-driven processes will further intensify flood risks, posing escalating threats to this climate-sensitive region under continued global warming. Our findings reveal a fundamental yet overlooked mechanism linking subseasonal variability to emerging hydroclimatic extremes in a warmer world.

## Teaser

Changes in monsoon rhythms are driving more extreme floods in South Asia’s historically dry regions.

## INTRODUCTION

Summer monsoon rainfall is a critical component of the South Asian climate system, underpinning agricultural productivity and ecosystem resilience (*1*). The spatial distribution of monsoon precipitation in South Asia exhibits a pronounced east-west asymmetry, with rainfall predominantly concentrated in the eastern and central regions, while northwestern South Asia (NWSA), encompassing Pakistan and northwest India, remains a typical semiarid zone (*2*, *3*). In recent decades, monsoon rainfall characteristics have shifted, with persistent heavy rainfall events—key drivers of catastrophic flooding—becoming more frequent and intense over NWSA (*4*, *5*). For example, the 2022 Pakistan floods caused thousands of fatalities, resulted in an estimated US $40 billion in damages, and led to a national emergency. The sudden onset of persistent heavy rainfall and severe flooding poses a greater threat to semiarid regions like NWSA than to the wetter core monsoon regions, where people and socioeconomic systems are more resilient to heavy rainfall. Identifying the underlying drivers and estimating their future changes in response to anthropogenic greenhouse warming are critical for developing more effective mitigation and adaptation strategies in this increasingly vulnerable region.

Many studies have linked the increasing persistent heavy rainfall and flooding to changes in the monsoon circulation driven by nonuniform surface warming (*4*, *6*–*9*). As Central Asia has undergone notable warming over the past two decades, meridional temperature gradient anomalies have shifted the cross-equatorial low-level southwesterly jet poleward, displacing the monsoon rain belt from central India toward NWSA (*4*). Meanwhile, the enhanced land-sea temperature contrast (*6*) and Indo-Pacific sea surface temperature anomalies (*7*) have strengthened monsoon flow, increasing rainfall across South Asia. At mid-to-high latitudes, a phase shift in the Silk Road teleconnection observed since the 1990s has induced upper-atmospheric divergence over NWSA, promoting heavier rainfall (*8*). In addition, the westward shift of local upward motion associated with the Hadley circulation, together with the westward displacement of the North Pacific Subtropical High, has further facilitated moisture transport toward NWSA and intensified rainfall (*9*, *10*). While these studies have provided valuable insights into changes in the evolving seasonal mean background conditions favoring increased rainfall, their focus on long-term circulation changes limits their ability to fully explain the mechanisms triggering persistent heavy rainfall and flood events, which typically persist for days rather than an entire season (*11*).

Between the seasonal mean state and synoptic-scale disturbances, intraseasonal oscillation (ISO) is the dominant mode of subseasonal variability in the global atmosphere, modulating weather systems through its phase evolution on a 10- to 90-day timescale (*12*–*14*). During the boreal summer, the tropical ISO exhibits vigorous activity over the Asian monsoon region, commonly called the monsoon ISO (*12*). The monsoon onset and the transition into an active rainy phase are often accompanied by the convective and cyclonic anomalies of monsoon ISO propagating from the equatorial Indian Ocean toward the Indian subcontinent (*15*, *16*). Historical heavy rainfall events over NWSA have also been linked to enhanced tropical ISO activity (*17*–*19*). In addition to monsoon ISO, mid-latitude Rossby waves and the associated teleconnections exhibit significant intraseasonal variability, which we refer to as the mid-latitude ISO in this study. By modulating vertical motion anomalies over NWSA, the upper-level ISO wave train can influence surface weather conditions, including rainfall variability over NWSA (*18*–*21*).

Despite the recognized links between ISO and rainfall variability in NWSA, the mechanisms through which ISO modulates persistent heavy rainfall and flood risks in this region remain poorly understood. In particular, it is essential to determine whether, and to what extent, the increasing threat of NWSA floods can be attributed to changes in tropical and mid-latitude ISOs (*22*–*25*), alongside contributions from mean moistening resulting from the Clausius-Clapeyron relationship (*26*) and seasonal background changes. Critically, these factors are expected to evolve differently under anthropogenic greenhouse gas warming. How tropical and mid-latitude ISOs will behave in the future, and how such changes will affect flood occurrence and severity, remain urgent questions for climate adaptation. By combining observations, reanalysis data, and Coupled Model Intercomparison Project Phase 6 (CMIP6) historical simulations and projections, this study identifies and quantifies the distinct roles of tropical and mid-latitude ISOs in driving persistent heavy rainfall and flood events over NWSA. This approach provides a previously overlooked perspective on how changes in ISO behavior shape hydroclimatic extremes and offers important implications for improving flood prediction and informing adaptation strategies in this highly vulnerable region.

## RESULTS

### Changes in summer rainfall and flooding over NWSA

**Fig. 1.**
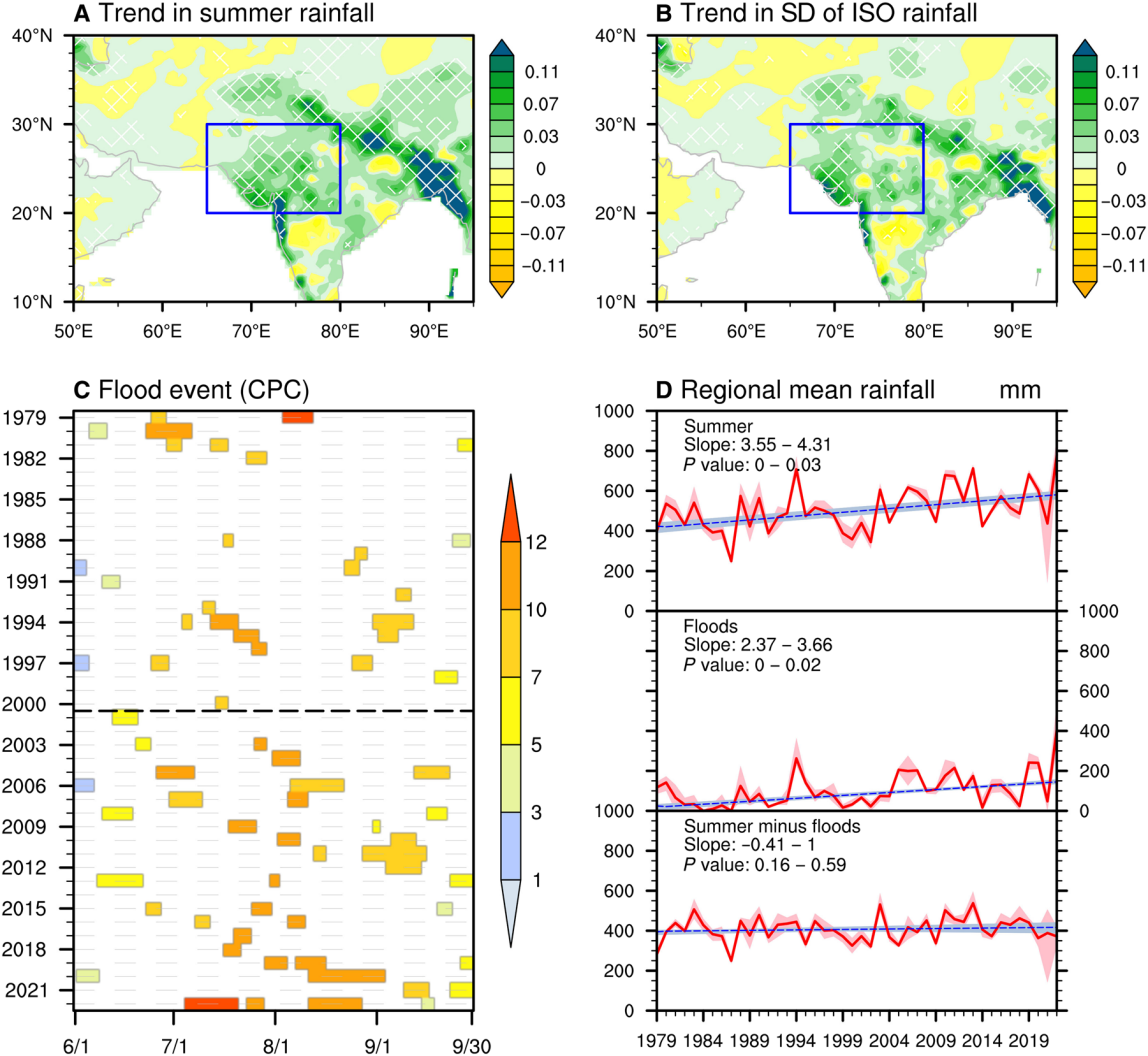
Increasing rainfall and flooding events over NWSA. (**A**) Spatial distribution of the linear trend in summer (June to September) rainfall (shading; unit: mm day^−1^ year^−1^) for 1979 to 2022 based on the CPC dataset. Hatched areas indicate statistically significant trends at the 95% confidence level. The blue box outlines NWSA. (**B**) Same as (A) but for trends in intraseasonal rainfall variability, defined by the standard deviation (SD) of 10- to 90-day rainfall during summer. (**C**) Historical duration (bar length) and intensity (bar color) of individual flood events, measured by the WAP index (unit: mm day^−1^) in Materials and Methods. The *x* axis represents months, while the *y* axis denotes years. (**D**) Time series of NWSA-averaged summer rainfall: (top) total summer rainfall, (middle) rainfall during flood periods, and (bottom) rainfall during nonflood periods (units: mm). Red lines show the ensemble mean from four datasets (CPC, ERA5, MERRA-2, and MSWEP), with shading indicating the interdataset range. Blue dashed lines denote corresponding linear trends, with trend slopes and associated *P* value ranges obtained on the basis of the different datasets shown in the top left corner. Trends were calculated and tested using the Sen-slope estimate and Mann-Kendall test, respectively.

The long-term trends in summer-mean rainfall amounts and intraseasonal rainfall variability over South Asia from 1979 to 2022 are shown in Fig. 1 (A and B), respectively. The climatologically wet regions and windward slopes of mountain ranges, such as the coastal areas along the Bay of Bengal, the southern edge of the Tibetan Plateau, and southwestern India, exhibit the most significant increases in both seasonal rainfall and intraseasonal variability (*22*). The semiarid regions of NWSA also show notable increases in monsoon rainfall and intraseasonal variability. The enhanced variability of rainfall at the intraseasonal timescale indicates a growing amplification of rainfall anomalies, suggesting that extreme rainfall and its associated severe flood events have become more pronounced over NWSA (Fig. 1, C and D, and figs. S1 and S2).

Flood activity over the past four decades, quantified using the weighted average of precipitation (WAP) index (Materials and Methods), indicates a marked intensification of flood events after 2000, with floods becoming stronger and more persistent (Fig. 1C). Both flood days and duration in NWSA exhibit statistically significant positive trends (fig. S1). The increasing occurrence of flood events is the primary driver of the positive trend in summer-mean rainfall amounts (Fig. 1, A and D). A significant upward trend is observed in flood-related rainfall (Fig. 1D, middle panel). When this flood-related component is removed from the total rainfall, the remaining component shows no significant trend (Fig. 1D, bottom panel). This result underscores that the increasing summer rainfall threat is primarily linked to flood events associated with persistent heavy rainfall, rather than changes in seasonal mean monsoon conditions. This finding remains robust across different datasets and under varying flood index parameter settings (fig. S2).

The consistent positive trends in intraseasonal rainfall variability and flood intensity indicate a strong link between ISOs and persistent heavy rainfall events over NWSA, aligning with the results of previous studies (*17*–*19*). Beyond long-term trends, ISO phase locking with flood occurrence is evident in recent cases (1998, 2010, and 2022; fig. S3). Each of these extreme events was associated with persistent heavy precipitation occurring during the convective phases of the ISO, highlighting its critical role in not only triggering floods but also modulating their intensity and duration. In addition to ISOs, mean conditions (annual cycle), low-frequency background (>90 days), and synoptic disturbances (<10 days) also exhibited positive anomalies or enhanced variability during flood events (fig. S3). The subsequent analysis quantifies the relative contributions of these different timescale components and clarifies how changes in tropical and mid-latitude ISOs drive the evolving characteristics of flood events.

### Intensified floods driven by enhanced northward propagation of the tropical ISO

**Fig. 2.**
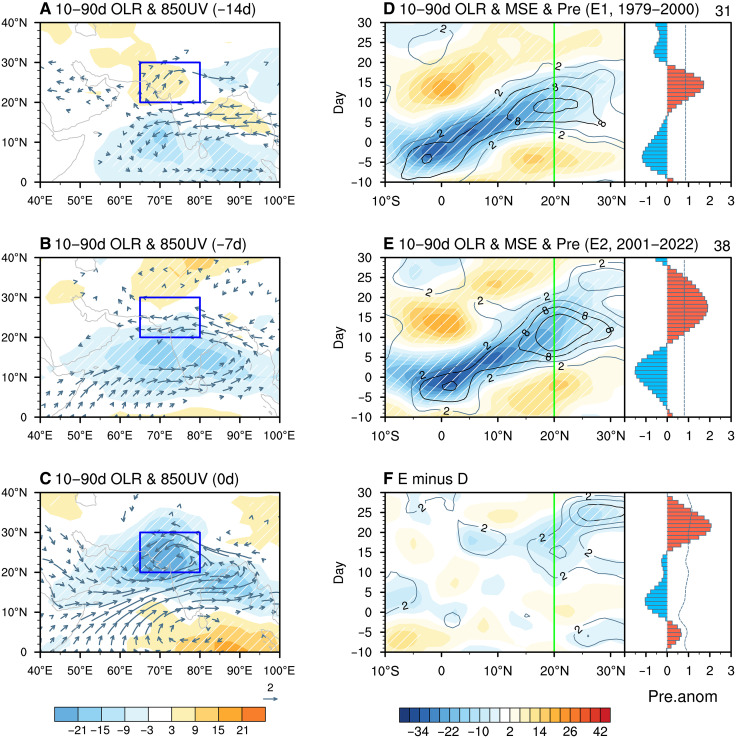
Changes in the monsoon ISO over the past four decades. (**A** to **C**) Composites of 10- to 90-day (d) OLR anomalies (shading; units: W m^−2^) and 850-hPa wind anomalies (vectors; units: m s^−1^; only statistically significant anomalies at the 95% confidence level are displayed) at 14, 7, and 0 days before flood events over NWSA, respectively. Blue boxes indicate the NWSA domain. (**D**) (Left) Latitude-time diagram of composited 10- to 90-day OLR (shading; units: W m^−2^) and moist static energy (MSE) anomalies (contours; units: 10^6^ J m^−2^) averaged over 65°E to 80°E, associated with tropical ISO events during the earlier epoch (E1: 1979 to 2000). The white dashed and black solid contours indicate regions where the anomalies are statistically significant at the 95% confidence level. The *x* axis represents latitude, and the *y* axis represents time, with day 0 marking the peak amplitude of the monsoon ISO at the equatorial region (5°S to 5°N). (Right) Temporal evolution of the corresponding 10- to 90-day rainfall (Pre) anomalies over NWSA (units: mm day^−1^). The light blue dashed line denotes the 95% confidence threshold based on Monte Carlo resampling. The number of tropical ISO cases is indicated in the top right corner. (**E** and **F**) Same as (D) but for the recent epoch (E2: 2001 to 2022) and the difference between E2 and E1. Green vertical lines in (D) to (F) mark the southern boundary latitude of NWSA.

To illustrate how the tropical ISO influences flooding over NWSA, we performed a composite analysis of intraseasonal (10- to 90-day) convection activity at 14, 7, and 0 days before flood occurrence (Fig. 2, A to C). About 2 weeks in advance, convective ISO signals appear over the equatorial Indian Ocean, while suppressed convection dominates NWSA (Fig. 2A). As the event progresses, convective anomalies propagate northward from the ocean toward the land areas (Fig. 2B). Flooding occurs when the maximum convective center of the ISO reaches NWSA, intensifying rainfall extremes (Fig. 2C). This northward propagation of the tropical ISO is consistent with previous findings by Chen *et al.* (*19*) and Xie *et al.* (*21*), who identified similar ISO-related mechanisms in their composite analyses of the 2022 Pakistan floods and other historical heavy rainfall events.

To determine whether and how changes in the tropical ISO have contributed to the increasing flood risks, we compare northward-propagating and non–northward-propagating ISO events using *K*-means clustering (Materials and Methods). Over the past four decades, 102 tropical ISO events were objectively classified into 69 northward-propagating and 33 non–northward-propagating events (fig. S4). The latter exhibited little association with rainfall over NWSA, underscoring the critical role of the northward-propagating ISO in modulating extreme rainfall and flood risks in the region. To further examine the role of the northward-propagating ISO in the flood evolution, we analyze its phase evolution and associated rainfall anomalies over NWSA during two periods: E1 (1979 to 2000) and E2 (2001 to 2022) (Fig. 2, D to F). The year 2000 was chosen as the dividing line to ensure statistical comparability and reliability between the two epochs of equal length. This choice is justified by the fact that NWSA flood characteristics exhibit a gradual increasing trend (Fig. 1) without any statistically significant regime shift or abrupt change point (not shown).

During E2, 38 northward-propagating ISO events occurred, an increase from 31 events in E1, indicating a higher frequency of northward ISO activity in recent decades. Beyond this frequency change, the latitudinal extent of ISO propagation also exhibited notable differences. In E1, northward propagation of the ISO tended to stagnate around 20°N (Fig. 2D, blue shading), resulting in a persistent rainfall anomaly over NWSA that developed 7 to 19 days after the ISO convection moved away from the equator (Fig. 2D, red bars from day 7 to day 19). In contrast, during E2, ISO signals continued propagating northward beyond 20°N (Fig. 2E, blue shading), leading to longer-lasting (day 9 to day 28) and more intense rainfall anomalies over NWSA (Fig. 2E, red bars). The direct comparison between E1 and E2 (Fig. 2F and fig. S5) clearly showcases the enhanced northward penetration of the tropical ISO deeper into NWSA, reinforcing persistent heavy rainfall (Fig. 2F, red bars).

Why does the tropical ISO exhibit an enhanced northward propagation in E2? Previous studies suggest that background thermodynamic and dynamic factors are crucial in shaping tropical ISO evolutions (*27*–*34*). In E2, warmer sea surface temperatures in the northern Arabian Sea (10°N to 20°N) (fig. S6A, purple box) moisten the lower troposphere, while an amplified land-sea temperature contrast strengthens cross-equatorial southwesterlies (fig. S6B, vectors). Combined with enhanced convective instability over 10°N to 30°N (fig. S6C), these conditions intensify ISO disturbances (*23*, *35*). CMIP6 historical and piControl simulations further confirm that elevated background moisture in the northern Arabian Sea correlates with stronger monsoon ISO over NWSA (fig. S6D). Increased background moisture strengthens the tropical ISO and creates a more favorable environment for continued northward development into NWSA (fig. S6, E to G). To further quantify these processes and link them to floods, we analyzed the relationships between background conditions, ISO activity, and flood metrics using scatter diagrams during 1979 to 2022 (fig. S7). The results show a clear sequence: A stronger land-sea temperature contrast intensifies southwesterlies over the Arabian Sea (fig. S7A), which in turn enhances low-level convergence and moisture accumulation over the northern Arabian Sea and NWSA (fig. S7, B and C). The resulting moistening favors more rigorous convection, leading to stronger ISO variability (fig. S7D), which is linked to higher flood frequency (fig. S7E) and, ultimately, greater flood rainfall (fig. S7F). These relationships are statistically significant, providing robust evidence that background thermodynamic and dynamic changes not only promote deeper ISO penetration into NWSA but also amplify flood risk through a coherent chain of processes.

### Prolonged flooding events linked to slower propagation of the mid-latitude ISO

**Fig. 3.**
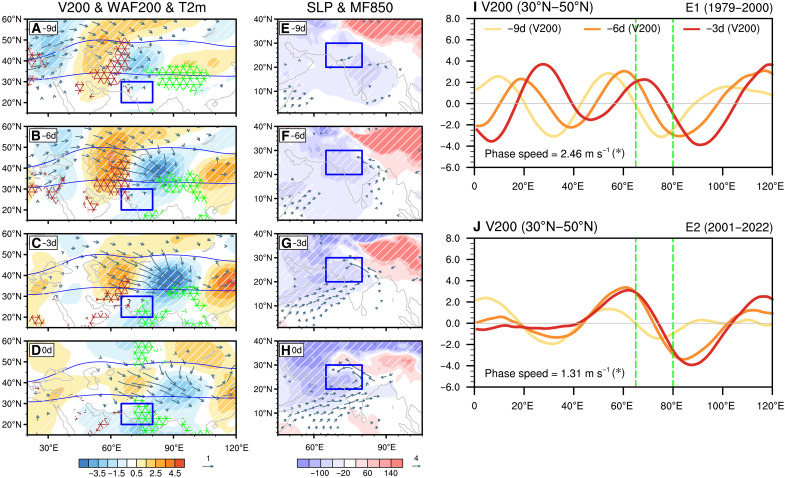
Changes in the mid-latitude ISO over the past four decades. (**A** to **D**) Composites of 200-hPa meridional wind anomalies (V200; shading; units: m s^−1^), 200-hPa WAF (WAF200; vectors; units: m^2^ s^−2^; only values >0.2 m^2^ s^−2^ are shown), and 2-m air temperature anomalies (T2m; red crosses denote positive anomalies, and green crosses denote negative anomalies; units: K; only anomalies significant at the 90% confidence level are shown) at 9, 6, 3, and 0 days before NWSA flood events. Regions with white dashed indicate anomalies significant at the 90% confidence level. Blue curves indicate the westerly jet position, marked by the June to September (JJAS)–averaged 200-hPa zonal wind (U200) threshold of 18 m s^−1^. (**E** to **H**) Similar to (A) to (D) but for the distributions of 10- to 90-day sea-level pressure anomalies (SLP; shading; units: Pa) and 850-hPa moisture flux anomalies (MF850; vector; units: 10^−3^ kg kg^−1^ m s^−1^; only anomalies significant at the 90% confidence level are shown) derived from flood events exclusively influenced by the mid-latitude ISO (Materials and Methods). The NWSA domain is outlined by blue. (**I**) Composites of V200 along the jet (30°N to 50°N) at 9 (yellow), 6 (orange), and 3 (red) days before flood events during E1 (1979 to 2000). The propagation speed of the intraseasonal wave train is indicated in the lower left corner, and single asterisks indicate that the composite value is statistically significant at the 90% confidence level. (**J**) Same as (I) but for the E2 period (2001 to 2022). Green vertical dashed lines in (I) and (J) indicate the longitudinal location of NWSA.

In addition to the tropical ISO, mid-latitude intraseasonal wave packets along the jet stream (Fig. 3, A to D, shading) also enhance heavy rainfall over NWSA. About 9 days before flood onset, a quasibarotropic and slowly southeastward-propagating mid-latitude ISO wave train develops (Fig. 3A). As this wave packet approaches NWSA, a pronounced dipole of near-surface warming (red crosses) and cooling (green crosses) emerges across the Iranian Plateau–Arabian Peninsula and the Tibetan Plateau (Fig. 3, A to D). Temperature-budget diagnostics indicate that this dipole is generated primarily by horizontal warm-air advection associated with the ISO-related southerly anomaly on the west and divergence-induced ascent and adiabatic cooling on the east (fig. S8).

To further isolate the mid-latitude influence, we composite the large-scale circulation using only flood events dominated by mid-latitude ISO activity (Materials and Methods; figs. S9 to S11). Consistent with the thermal dipole, the resulting circulation anomalies (Fig. 3, E to H) reveal a coherent low-pressure anomaly to the northwest of NWSA and a high-pressure anomaly to the northeast, together forming a pressure dipole characteristic of the mid-latitude ISO. This dipole organizes two moisture-transport pathways into NWSA: Southeasterly flow along the southern flank of the high-pressure anomaly channels moisture from the Bay of Bengal, while southwesterly flow associated with the low-pressure anomaly transports moisture from the Arabian Sea (vectors in Fig. 3, E to H). These features indicate that mid-latitude ISOs can precondition the environment for heavy rainfall by strengthening both zonal and meridional moisture fluxes. The Geophysical Fluid Dynamics Laboratory Low Ocean Atmosphere Resolution (LOAR) sensitivity experiments further support this mechanism (Materials and Methods; fig. S12). The control run reproduces the observed mid-latitude ISO wave trains, pressure dipole, and moisture transports (fig. S12, A to H), and these features remain, although weaker, when tropical ISOs are suppressed in the LP90 experiment (fig. S12, I to L). Thus, mid-latitude ISOs can independently modulate NWSA heavy rainfall, complementing but not requiring concurrent tropical variability.

Over the past four decades, the characteristics of the mid-latitude ISO have shifted in a manner that further amplifies flood risk. During E2, this slow-propagating feature became even slower (1.31 m s^−1^; Fig. 3J) compared to E1 (2.46 m s^−1^; Fig. 3I). This further prolonged the residence time of ISO-related circulation and moisture anomalies over NWSA, extending the duration of flood events. In addition, in E1, the ISO wave packet tended to decay more rapidly as it approached NWSA (Fig. 3I, yellow, orange, and red curves; fig. S13, left panels). In contrast, during E2, the amplitude of the wave train was sustained throughout the flood period (Fig. 3J, color curves; fig. S13, right panels), continuously reinforcing favorable conditions for prolonged precipitation. The slowing of the mid-latitude ISO propagation is a robust feature, consistently observed even when applying different methods to detect the wave packet (fig. S14), further confirming its role in extending the duration of extreme rainfall events over NWSA.

The slower propagation of mid-latitude ISOs is closely linked to the weakening of the Eurasian jet stream over the past four decades (*36*–*38*). Because the background westerlies act as a waveguide for mid-latitude disturbances (*39*), a weaker jet leads to slower ISO propagation (fig. S15). To further verify this linkage, we expanded the analysis to CMIP6 historical simulations and examined the relationship of both jet intensity and jet latitude to mid-latitude ISO periodicity. The multimodel results show a significant negative correlation between jet intensity and ISO periodicity (fig. S15D), while no meaningful relationship is found with jet latitude (fig. S15H). Observations further reveal a clear long-term weakening of the jet but no robust trend in its meridional position (fig. S16). These results demonstrate that jet weakening, rather than meridional displacement, is responsible for the deceleration of mid-latitude ISO propagation, thereby prolonging its influence over NWSA and sustaining persistent extreme rainfall events (Fig. 3).

### Quantifying the relative contributions of tropical and mid-latitude ISOs to increased NWSA floods

**Fig. 4.**
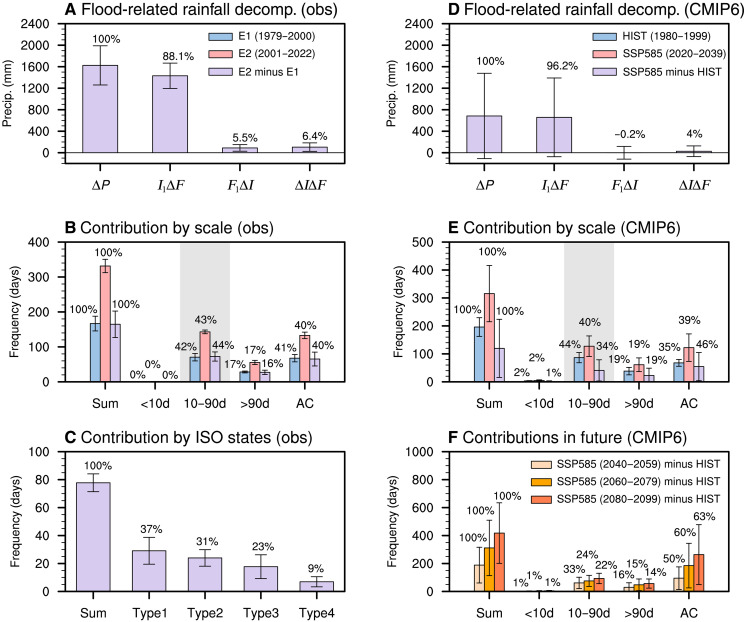
Quantitative contributions of ISOs to long-term changes in floods. (**A**) Relative contributions of changes in flood frequency ($I_{1}∆F$), flood intensity ($F_{1}∆I$), and their interaction (∆I∆F) to the increase in NWSA flood-related rainfall during E2 versus E1 (∆P). Units: mm. Whiskers indicate ±1 SD across different rainfall datasets. (**B**) As in (A) but showing the relative contributions of different timescale components to flood-day occurrence during E1 (blue), E2 (pink), and their differences (purple). From left to right: total flood days and contributions from <10-day, 10- to 90-day, >90-day, and annual cycle (AC) components. Values above bars show contributions of each component to the total. Gray shading highlights the contributions from the 10- to 90-day ISO. (**C**) As in (B) but for the relative contributions of different ISO states to the increase in flood days. From left to right: total flood days associated with ISOs (identical to the third purple bar in B), concurrent tropical and mid-latitude ISOs, tropical ISO only, mid-latitude ISO only, and neither tropical nor mid-latitude ISOs. Whiskers indicate the SD across different datasets and definitions, as described in Materials and Methods. (**D** and **E**) Same as (A) and (B) but showing quantitative assessments from CMIP6 historical (HIST) simulations (1980 to 1999; blue), SSP5-8.5 high-emission scenario projections (2020 to 2039; pink), and their differences (purple). Whiskers denote ±1 SD across 27 models. (**F**) As in the purple bars of (E) but extended to future periods 2040 to 2059 (light orange), 2060 to 2079 (orange), and 2080 to 2099 (dark orange), each relative to the historical period.

Having established the increase in NWSA flood events over the past four decades (Fig. 1) and the associated changes in tropical and mid-latitude ISOs (Figs. 2 and 3), we next quantify the extent to which ISO behavior contributed to this increase. As a first step, we decomposed the overall rise in flood-related rainfall (Fig. 1D) into three components: $I_{1}∆F$, representing the effect of changes in flood frequency; $F_{1}∆I$, representing the effect of changes in flood intensity; and ∆I∆F, representing the nonlinear interaction between frequency and intensity. Here, I is the average intensity of flood events (flood-related rainfall divided by the number of flood days per event), F is the frequency of flood days, ∆ denotes the difference between E2 (2001 to 2022) and E1 (1979 to 2000) (E2 minus E1), and the subscript 1 refers to E1. This analysis indicates that 88% of the increase in flood-related rainfall is attributable to more frequent flood days, while changes in intensity and nonlinear effects contribute only ~5 to 6% each (Fig. 4A). In other words, the increase mainly reflects a greater frequency of the daily rainfall exceeding the extreme threshold used to define a flood event.

Second, we estimated the relative roles of different timescale variability components using a Shapley-based attribution approach. In this framework, the probability of daily WAP index exceeding the flood threshold is partitioned among synoptic (<10 days), intraseasonal (10 to 90 days), low-frequency (>90 days), and annual cycle (climatological daily mean) components. This approach ensures that contributions are assigned consistently and independently of the order in which components are considered (Materials and Methods). The results indicate that ISO variability accounts for ~42% of flood occurrence during E1, ~43% during E2, and ~44% of the epochal increase (Fig. 4B), comparable to but slightly higher than the contributions associated with changes in the mean state (~40%). The >90-day component, reflecting interannual to decadal modes, also contributes positively but modestly (~16%). By contrast, the negligible contribution from synoptic disturbances arises because high-frequency variability tends to cancel between positive and negative anomalies during flood events (fig. S3).

To disentangle the relative roles of different ISO sources on NWSA floods, we classified flood days into four types: (i) both tropical and mid-latitude ISOs present, (ii) tropical ISO only, (iii) mid-latitude ISO only, and (iv) neither tropical nor mid-latitude ISO detected. The definitions of these four states, on the basis of geographic location, propagation characteristics, and intensity, are provided in Materials and Methods (figs. S9 to S11). Probability analysis indicates that more than 90% of the increase in flood occurrence is associated with changes in ISO activity—37% from concurrent tropical and mid-latitude ISOs, 31% from tropical ISOs alone, and 23% from mid-latitude ISOs alone. The remaining ~9% likely reflects local 10- to 90-day variability not linked to organized or persistent propagation from either the tropics or the mid-latitudes (Fig. 4C).

**Fig. 5.**
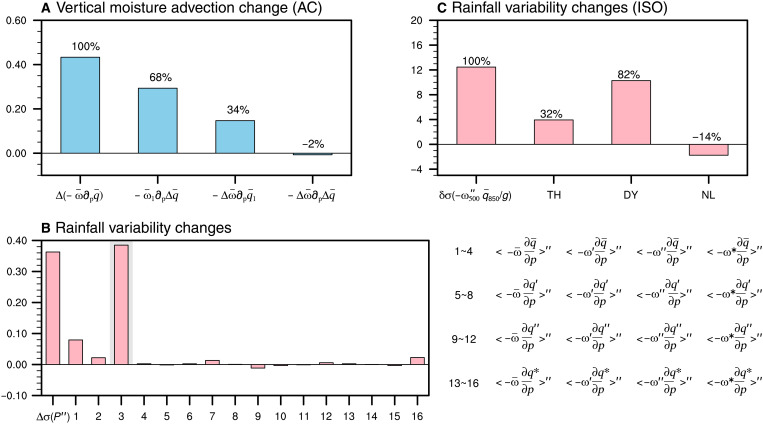
Effects of global warming on mean rainfall and intraseasonal variability. (**A**) Decomposition of the decadal change of mean advection of the mean moisture on the basis of the diagnosis of Eq. 3 (units: 10^−5^ kg m^−2^ s^−1^). From left to right: change in mean rainfall, the thermodynamic effect of mean-moisture changes with circulation fixed, the dynamic effect of circulation changes with moisture fixed, and their nonlinear interaction. Values above bars show contributions of each component to the total. (**B**) Differences in the SD of intraseasonal rainfall between the two periods (leftmost bar; units: mm day^−1^), while the right bars represent 16 decomposed components on the basis of vertical moisture advection (right bars; units: 10^−5^ kg m^−2^ s^−1^), corresponding to Eq. 4. The gray shading indicates the term with the largest contribution. (**C**) Percentage changes (%) in the variability of 10- to 90-day vertical moisture advection and its thermodynamic (TH), dynamic (DY), and nonlinear (NL) components over NWSA, as defined in Eq. 5.

Building on these attribution results, we next investigate the physical processes responsible for the increase in mean rainfall and the amplification of ISO rainfall variability that lead to more frequent exceedance of the flood threshold using a scale-decomposed moisture-budget analysis (Eqs. 3 to 5 in Materials and Methods; Fig. 5). The upward shift in mean rainfall from E1 to E2 is largely explained by enhanced mean moisture (~68%), even if circulation remains unchanged, with circulation changes contributing a smaller share (~34%) and nonlinear interactions playing only a minor role (Fig. 5A). By contrast, the growth of ISO rainfall variability stems mainly from variations in ISO circulation interacting with mean moisture, whereas other terms make little contribution (Fig. 5B). A further separation shows that this variability is driven mainly by stronger ISO circulation variability, with mean moistening alone providing a smaller but positive contribution (Fig. 5C). Overall, these results indicate that both global warming–driven moistening and changes in ISO circulation increase the likelihood of rainfall exceedance, with the former providing a broad thermodynamic background and the latter acting as the dynamic trigger that amplifies flood occurrence over NWSA. Nevertheless, the increase in flood-related rainfall amounts is dominated by mean-state changes (fig. S17, A and B), consistent with the fact that ISO-related rainfall anomalies represent perturbations whose amplitudes are substantially smaller than the climatological mean.

### Escalating NWSA flood risks by future ISO changes under continued greenhouse warming

**Fig. 6.**
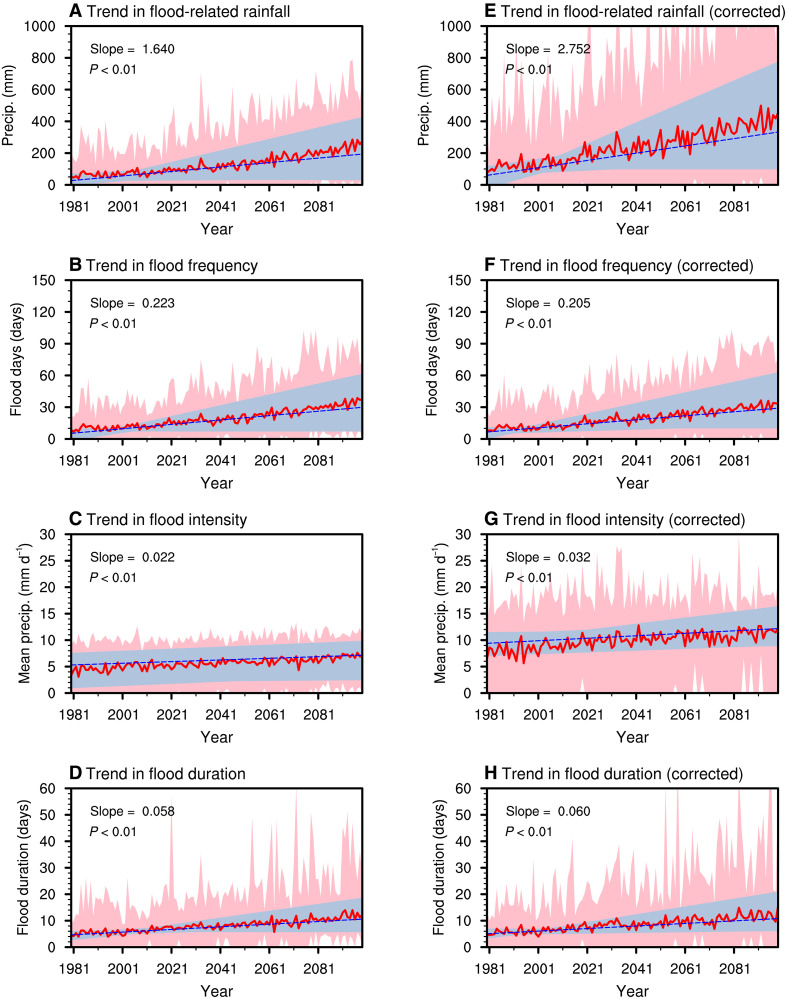
Future changes in NWSA flood characteristics. (**A**) Time series of flood-related rainfall over NWSA from 1980 to 2099, derived from CMIP6 historical simulations and SSP5-8.5 high-emission scenario projections. The red curve (unit: mm) represents the multimodel ensemble mean of flood-related rainfall, while the blue line (unit: mm year^−1^) indicates the linear trend, with shading indicating the spread of the model simulations. The trend slope and its *P* value are shown in the top left corner. (**B** to **D**) Same as (A) but for the frequency of flood events (units: days), the average intensity of flood-related rainfall (unit: mm day^−1^), and the duration of NWSA flood events (units: days), respectively. (**E** to **H**) As in (A) to (D) but using the outputs of NEX-GDDP-CMIP6 dataset.

**Fig. 7.**
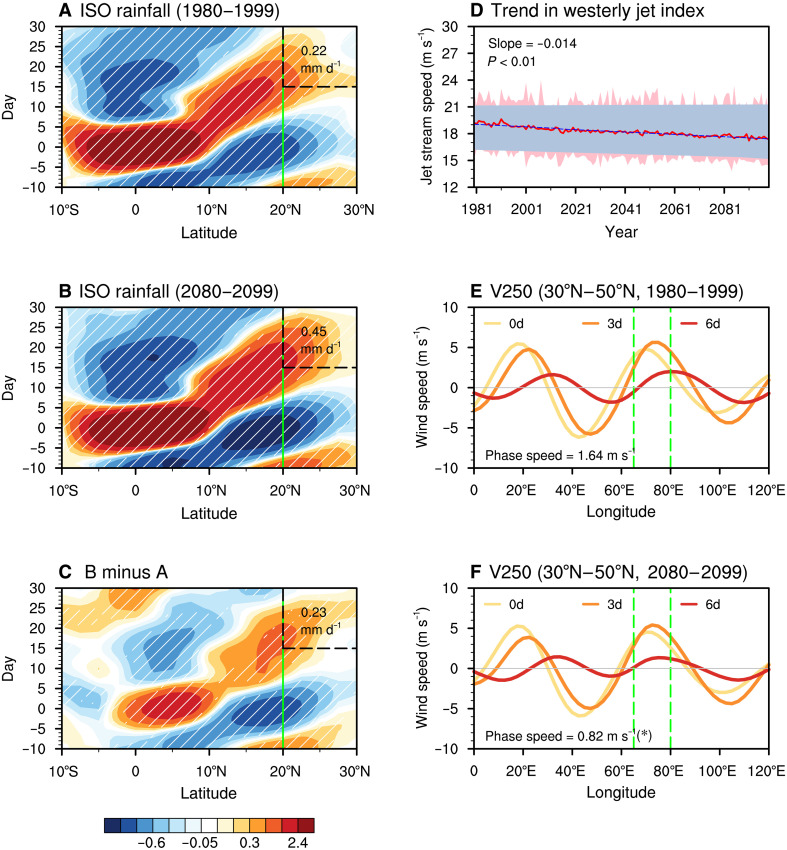
Future changes in tropical and mid-latitude ISO activity. (**A**) Latitude-time evolution of tropical ISO-related rainfall (units: mm day^−1^) during 1980 to 1999 on the basis of the historical run, represented by 10- to 90-day precipitation fields regressed onto the standardized time series of 10- to 90-day precipitation over the equatorial Indian Ocean (65°E to 80°E, 5°S to 5°N). Hatched areas indicate regions where at least 66% of the models agree on the sign of the changes. Green vertical lines mark the southern boundary of NWSA. The black dashed box highlights ISO-related rainfall over NWSA, with its area-averaged value displayed in the corner. (**B** and **C**) Same as (A) but for tropical ISO-related rainfall during 2080 to 2099 on the basis of the SSP5-8.5 warming scenario projection and the difference between 2080 to 2099 and 1980 to 1999, respectively. (**D**) Same as Fig. 6A but for the westerly jet index. (**E** and **F**) Same as Fig. 3I but for the composite results of mid-latitude ISO events in CMIP6 simulations during 1980 to 1999 (historical run) and 2080 to 2099 (SSP5-8.5 warming scenario projection), respectively.

The increasing trend in NWSA flood activity over the past four decades is projected to persist under continued greenhouse warming. Under the high-emission (SSP5-8.5) scenario, a CMIP6 multimodel ensemble with realistic simulations of ISO and jet patterns reveals significant increasing trends in flood-related rainfall, flood frequency, and average intensity from 1980 to 2099 (Fig. 6, A to C). To confirm the robustness of projected changes in NWSA flood characteristics, we further analyzed flood events using the bias-corrected, high-resolution NEX-GDDP-CMIP6 dataset. Significant positive trends in flood risk are evident (Fig. 6, E and G), with steeper slopes in flood rainfall and intensity after quantile-based correction. Because flood detection relies on relative thresholds across datasets, the projected frequency of flood cases remains consistent between corrected and raw projections (Fig. 6F). These results highlight the value of bias correction in improving the representation of rainfall amplitudes while also confirming the robustness of the projected changes in flood characteristics. The escalating flood risks (Fig. 6) are partly linked to an intensifying, northward-propagating tropical ISO, which enhances convective development and rainfall extremes over the NWSA region under future greenhouse warming. Relative to the present-day climate, the tropical ISO exhibits substantially stronger amplitude from the equatorial region to NWSA in a warmer world (Fig. 7, A to C). By the end of the century, the associated tropical ISO-induced precipitation anomalies over NWSA nearly double compared to those in the late 20th century (Fig. 7, A to C, black dashed boxes).

Beyond the intensification of the tropical ISO, the mid-latitude ISO is projected to propagate more slowly, potentially prolonging flood duration (Fig. 6, D and H). This slowdown could be attributed to the weakening of subtropical jet streams over Eurasia under a future warming scenario (Fig. 7D). CMIP6 model simulations indicate that the phase speed of ISO wave packets will decrease from ~1.64 m s^−1^ in the late 20th century (Fig. 7E) to 0.82 m s^−1^ by the end of the 21st century (Fig. 7F). Together, these findings suggest that under very-high-emission scenarios, the synergistic combination of stronger tropical ISO convection and slower mid-latitude ISO propagation will significantly amplify flood risks in NWSA, increasing the region’s vulnerability to prolonged and extreme flooding throughout the 21st century.

To quantify how changes in ISOs under SSP5-8.5 warming contribute to increasing flood risk, we applied the same Shapley attribution framework used for the observational analysis to CMIP6 projection data (Fig. 4, D to F). In the near term (2020 to 2039), the increase in flood-related rainfall is dominated (~95%) by enhanced flood occurrence as a result of the higher likelihood of daily WAP index exceeding the flood threshold (Fig. 4D), consistent with the historical observational results (Fig. 4A). ISO activity continues to contribute positively to flood occurrence by increasing the probability of threshold exceedance, but its relative role decreases with time (Fig. 4F). In contrast, under the strong greenhouse gas forcing of the SSP5-8.5 scenario, atmospheric moistening intensifies substantially and favors more extreme rainfall (*26*, *40*). The effect of mean-state changes steadily strengthens and eventually becomes the dominant contributor (Fig. 4F). Quantitatively, the ISO contribution to flood frequency declines from 33 to 34% in the mid-21st century (2020 to 2059) to ~22% by the late 21st century (2080 to 2099), while the contribution to flood frequency of mean-state change increases from 46 to 50% to ~63%. A similar dominance of the mean state is found for flood-related rainfall amounts (fig. S17C), underscoring that changes in the mean background associated with thermodynamic moistening become the primary driver of heightened flood risks under future climate conditions.

## DISCUSSION

NWSA, historically a semiarid region, is undergoing a marked transition toward wetter conditions, with increasing extreme rainfall and flood events. Our findings show that long-term changes in both tropical and mid-latitude ISOs are critical yet often overlooked drivers of this transformation. Under global warming, enhanced northward propagation of the tropical ISO and slower southeastward propagation of mid-latitude ISOs create favorable conditions for more prolonged and intense flood events. Shapley attribution reveals that ISOs contributed ~44% to the increase in flood frequency over the past four decades and will continue to play a positive role through the end of the 21st century under strong SSP5-8.5 warming, although their contribution to flood frequency declines to ~22% as mean-state changes, dominated by atmospheric moistening, rise to ~63%. While ISOs exert a key dynamical influence on threshold exceedance and therefore on flood occurrence, the increases in flood-related rainfall amounts from the historical to future periods are dominated by mean moistening.

The physical mechanisms underlying these changes can be linked to broader climate change processes. The enhanced northward propagation of tropical ISOs is consistent with amplified land-sea thermal contrasts and increased moisture availability over South Asia under warming (*41*, *42*), which sustain higher moist static energy and allow convection to penetrate farther inland. In contrast, the slower propagation of mid-latitude ISOs is associated with a weakening of the subtropical jet, which prolongs the residence time of favorable circulation anomalies over NWSA. The weakening of the subtropical jet likely reflects multiple interacting influences. Basin-scale sea surface temperature variability in the Atlantic and Pacific Oceans can modify the jet by altering meridional temperature gradients and exciting large-scale Rossby wave trains (*43*–*45*). Additional contributing factors proposed in the literature include Arctic amplification, although its regional impacts remain mixed across seasons (*36*, *46*), and anthropogenic aerosol forcing, which has been identified as a dominant driver in some studies (*47*). The relative roles of these processes in shaping future jet changes remain an active area of research.

Hydroclimatic extremes increasingly challenge modern prediction systems and threaten climate resilience across critical sections, including infrastructure, agriculture, and ecosystems. Our findings establish a strong mechanistic linkage between the ISO and floods driven by persistent heavy rainfall, suggesting that the ISO is a crucial source of predictability for extreme rainfall events in NWSA, a finding that offers transformative potential for mitigating escalating flood risks. By incorporating ISO signals into dynamical and statistical prediction models, we can enhance early warning systems and extend actionable lead times for flood risk management beyond the 10-day limit of conventional weather forecasts (*48*), enabling proactive measures like reservoir management and agricultural planning—critical for a region where >60% of livelihoods rely on climate-vulnerable sectors. Furthermore, the growing application of artificial intelligence and machine learning techniques in climate prediction presents emerging opportunities for leveraging ISO-related patterns to improve flood prediction accuracy (*49*, *50*). Future advances that combine artificial intelligence–driven pattern recognition with ISO dynamics may hold promise for improving subseasonal-to-seasonal flood prediction in NWSA, a region where traditional numerical models struggle, yet the stakes of poor predictions are exceptionally high.

## MATERIALS AND METHODS

### Observation and CMIP6 datasets

Given that trend analysis is sensitive to data length, with larger samples ensuring more robust results, we used four long-term gridded daily precipitation datasets spanning the past four decades to reduce uncertainty: (i) the Multi-Source Weighted-Ensemble Precipitation (MSWEP) (*51*), (ii) the National Oceanic and Atmospheric Administration (NOAA) Climate Prediction Center (CPC) dataset (*52*), (iii) the Modern-Era Retrospective Analysis for Research and Applications version 2 (MERRA-2) (*53*), and (iv) the fifth-generation reanalysis from ECMWF (ERA5) (*54*). Convective activity was analyzed using daily outgoing longwave radiation (OLR) from NOAA (*55*). Large-scale thermodynamic and dynamic anomalies were examined using ERA5 and MERRA-2, including three-dimensional zonal and meridional winds (*u* and *v*), vertical velocity (ω), specific humidity (*q*), and geopotential height (*Z*) from 1000 to 100 hPa. The dataset periods and horizontal resolutions are as follows: MSWEP (1979 to 2022; 0.1° by 0.1°), CPC (1979 to 2022; 0.5° by 0.5°), MERRA-2 (1980 to 2022; 0.5° by 0.625°), and ERA5 (1979 to 2022; 1° by 1°). In this study, summer refers to June to September (JJAS).

For model simulations, we collected data from the piControl experiment, historical simulations (1979 to 2014), and future projections (2015 to 2100) under the SSP5-8.5 warming scenario from CMIP6 (*56*). Available fields, including rainfall and circulation data, from 27 models are listed in table S1, with all datasets regridded to a 2.5°-by-2.5° resolution. To further assess the impact of precipitation biases, we also used the high-resolution bias-corrected dataset, the NASA Earth Exchange Global Daily Downscaled Projections for CMIP6 (NEX-GDDP-CMIP6; 0.25° by 0.25°) (*57*), which includes both historical simulations and future projections. For fair comparison, only the models listed in table S1 were used.

### Definitions of flood events

Because of the lack of long-term flood observations at grid-cell scales in South Asia, we adopt the widely used WAP index (*58*) to characterize floods associated with heavy rainfall. This index considers both the immediate contribution of daily precipitation and the cumulative effect of antecedent rainfall. The WAP index is calculated as

$$\text{WAP}=\left(1-a\right)\sum_{n=0}^{N}a^{n}P_{n}$$ (1)

where $P_{n}$ represents the rainfall on the *n*-th day, a is a constant between 0 and 1, and *N* is the total number of days of antecedent rainfall considered. On the basis of the recommendations of the index developers, we set *a* = 0.9 and *N* = 7 to account for flooding caused by persistent heavy rainfall. A flood event is identified when the daily WAP index, calculated using the area-averaged rainfall over NWSA, exceeds the 90th percentile threshold and lasts for at least three consecutive days. The 90th percentile threshold is defined for each day using a 15-day moving window to account for the annual cycle of the local WAP index. Sensitivity tests confirm that the results are not significantly affected by the choice of window length.

### Shapley-based attribution of timescale contributions to flood occurrence

To quantify the relative contributions of different variability components to flood occurrence, we applied a Shapley value decomposition, originally developed in cooperative game theory (*59*). The daily WAP index was first separated into four components: the annual cycle, synoptic-scale (<10-day), intraseasonal (10- to 90-day), and lower-frequency (>90-day) variability. Specifically, daily climatology was removed to define the annual cycle, and the residual anomalies were decomposed using Lanczos filters (*60*). A 10- to 90-day bandpass filter isolated the intraseasonal component, while a 10-day high pass and a 90-day low pass captured the synoptic-scale disturbances and the lower-frequency background, respectively. This bandpass filtering approach effectively removes lower-frequency climate modes, such as ENSO (El Niño–Southern Oscillation), from the intraseasonal band, as the ISO signals remain nearly unchanged when ENSO-related rainfall variability is regressed out before bandpass filtering.

Within the Shapley framework, flood occurrence is treated as the “total outcome,” and each component is considered a “player” contributing to this outcome. For a given flood day, the payoff function is defined as whether the daily WAP index exceeds the flood threshold, set at the 90th percentile of the daily WAP distribution. The Shapley value of each component is computed as the average marginal contribution across all possible orders in which the components are added. The marginal contribution of a given player is defined as the difference in payoff when that player is added to a coalition. Formally, the Shapley value or component 𝑖 is

$$\phi_{i}=\sum_{S\subseteq N\backslash \left\{i\right\}}\frac{|S|!\left(|N|-|S|-1\right)!}{|N|!}\left[\upsilon \left(S⋃\left\{i\right\}\right)-\upsilon \left(S\right)\right]$$ (2)

where N = 4 (the set of all players), S is a subset of *N* not containing 𝑖, and υ(S) is the payoff function, which is defined as 1 if the daily WAP index based on components in S exceeds the flood threshold, and 0 otherwise. This averaging across all possible orderings ensures a fair and order-independent allocation of contributions. The Shapley decomposition therefore provides an objective measure of how much each timescale component contributes to the daily WAP index exceeding the flood threshold, both individually and in combination.

As a simple illustration, if each variability component alone is sufficient to exceed the threshold for trigger a flood, then the Shapley value for each component is equal (25%). If only the combination of the ISO and the mean state can produce a flood, then their Shapley values are 50% each, while the others are zero. If most combinations that include the mean state exceed the threshold, while those without it do not, then the mean state receives the largest share (50%), while the other three components contribute equally to the remainder (one-sixth each, ~16.7%).

### Clustering analysis

*K*-Means clustering was used to objectively classify the propagation characteristics of the ISO (*61*), following these steps. First, a tropical ISO event was selected when the area-averaged intraseasonal OLR over the equatorial Indian Ocean (65°E to 80°E, 5°S to 5°N) was active [<−1 standard deviation (SD)] for at least three consecutive days. The day with the minimum OLR value within the event period was defined as day 0. A total of 109 cases were identified during the 44 summers from 1979 to 2022. Next, cluster analysis was applied to classify distinct types of propagating patterns along the 65°E-to-80°E longitude band. To achieve this, Hovmöller diagrams spanning days −10 to 20 and covering 10°S to 30°N were analyzed for all 109 cases. Last, clustering was refined using silhouette values greater than 0.05, resulting in the identification of 69 northward-propagating events penetrating NWSA and 33 events with limited northward propagation (or that were non–northward-propagating), which were more prone to exhibiting stronger eastward-propagating components toward East Asia.

### Detection of tropical and mid-latitude ISO events

To distinguish the impacts of tropical and mid-latitude ISOs on floods, we identify their dominant 10- to 90-day propagating signals. The tropical ISO is a northward-propagating convective mode from the equatorial Indian Ocean to South Asia (40°E to 100°E, 10°S to 30°N), and the mid-latitude ISO is a southeastward-propagating wave train along the subtropical jet (0° to 120°E, 25°N to 55°N). Empirical Orthogonal Function (EOF) analysis is applied to the filtered anomalies, and the leading two EOFs form a quadrature pair that represents the propagating structure (fig. S9).

Floods related to ISOs are diagnosed using two complementary approaches. The first method is linked to the phase evolution of principal component (PC) series (figs. S10A and S11A). A flood is considered associated with the tropical (mid-latitude) ISO if, within the preceding 16 (10) days preceding a flood onset, PC2 (PC1) remains positive with amplitude >0.5 SD for at least 8 (5) days, while PC1 (PC2) undergoes a positive-to-negative (negative-to-positive) transition. Composites of events that capture propagating signals are shown in figs. S10B and S11B, while those without clear propagation are shown in figs. S10C and S11C. Sensitivity tests using alternative thresholds (±0.5 to 1 SD) and phase windows (±1 or 2 days) confirm robustness.

The second method projects flood-day anomalies onto climatological propagating ISO patterns derived from NWSA flood events (figs. S10D and S11D). For each flood event, Hovmöller diagrams of 10- to 90-day OLR (−25 to 0 days, latitude-time) and 200-hPa meridional wind (−15 to 0 days, longitude-time) anomalies were constructed to trace tropical and mid-latitude ISO evolution, respectively. An event is classified as propagating if its Hovmöller pattern shows a correlation with the climatological composite that exceeds the 95% confidence level based on 5000 Monte Carlo resampling. Composites of events that capture propagating signals are shown in figs. S10E and S11E, while those without clear propagation are shown in figs. S10F and S11F. Using this classification, we identified 19 flood events dominated solely by mid-latitude ISOs; these events form the basis of the composites shown in Fig. 3 (E to H).

### Moisture budget diagnosis for mean and ISO variability changes

From the moisture budget perspective, extreme precipitation events over NWSA can be approximated by the column-integrated vertical moisture advection (*21*). The change in mean precipitation ($∆\bar{P}$) between E2 and E1 is therefore written as

$$\begin{array}{cc} ∆\bar{P}\approx ∆< & -\bar{\omega }\frac{\partial \bar{q}}{\partial p}>\\ & =-<{\bar{\omega }}_{1}∆\left(\frac{\partial \bar{q}}{\partial p}\right)+∆\bar{\omega }\left(\frac{\partial {\bar{q}}_{1}}{\partial p}\right)+∆\bar{\omega }∆(\frac{\partial \bar{q}}{\partial p})> \end{array}$$ (3)

where the overbar denotes mean components derived from the climatological daily mean, ω is the vertical velocity (Pa s^−1^), q is specific humidity, and ⟨·⟩ represents vertical integration from surface to 100 hPa. Subscript 1 denotes the climatological state in E1, while ∆ represents the difference between E2 and E1 (E2 minus E1). The three terms on the right-hand side correspond to (i) the thermodynamic effect of mean-moisture changes with circulation fixed, (ii) the dynamic effect of circulation changes with moisture fixed, and (iii) their nonlinear interaction.

The change in ISO rainfall variability, measured by the SD σ, can be expressed as

$$\begin{array}{cc} ∆\sigma \left(P^{\prime \prime }\right) & \approx ∆\sigma \left(<-{\left(\omega \frac{\partial q}{\partial p}\right)}^{\prime \prime }>\right)\\ & \approx ∆\sigma \left(<-\left(\bar{\omega }\frac{\partial \bar{q}}{\partial p}+\omega^{\prime }\frac{\partial \bar{q}}{\partial p}+\omega^{\prime \prime }\frac{\partial \bar{q}}{\partial p}+\omega^{*}\frac{\partial \bar{q}}{\partial p}+\bar{\omega }\frac{\partial q^{\prime }}{\partial p}\right.\right.\\ & +\omega^{\prime }\frac{\partial q^{\prime }}{\partial p}+\omega^{\prime \prime }\frac{\partial q^{\prime }}{\partial p}+\omega^{*}\frac{\partial q^{\prime }}{\partial p}+\bar{\omega }\frac{\partial q^{\prime \prime }}{\partial p}+\omega^{\prime }\frac{\partial q^{\prime \prime }}{\partial p}+\omega^{\prime \prime }\frac{\partial q^{\prime \prime }}{\partial p}\\ & \left.{\left.+\omega^{*}\frac{\partial q^{\prime \prime }}{\partial p}+\bar{\omega }\frac{\partial q^{*}}{\partial p}+\omega^{\prime }\frac{\partial q^{*}}{\partial p}+\omega^{\prime \prime }\frac{\partial q^{*}}{\partial p}+\omega^{*}\frac{\partial q^{*}}{\partial p}\right)}^{\prime \prime }>\right) \end{array}$$ (4)

where ∆σ represents the SD change between E1 and E2. The asterisks, double primes, and single primes denote the <10-day, 10- to 90-day, and >90-day components, respectively. Because linear and nonlinear cross-timescale interactions can project onto the 10- to 90-day band, all combinations are included. We then focus on the dominant term—ISO vertical motion anomalies acting on mean moisture—whose change can be further decomposed into thermodynamic and dynamic effects (*62*)

$$\left\{\begin{array}{c} \delta \sigma \left(P^{\prime \prime }\right)\approx \delta \sigma \left(<-{\left(\omega^{\prime \prime }\frac{\partial \bar{q}}{\partial p}\right)}^{\prime \prime }>\right)\approx \delta \sigma \left(<-\left(\omega^{\prime \prime }\frac{\partial \bar{q}}{\partial p}\right)>\right)\\ \approx \delta \sigma \left(\frac{-\omega_{500}^{\prime \prime }{\bar{q}}_{850}}{g}\right)\approx \text{TH}+\text{DY}+\text{NL}\\ \text{TH}\approx \delta {\bar{q}}_{850}\\ \text{DY}\approx \delta \sigma \left(-\omega_{500}^{\prime \prime }\right) \end{array}\right.$$ (5)

where δσ represents the percentage change in SD with respect to the baseline period. $\omega_{500}^{\prime \prime }$ is the intraseasonal 500-hPa vertical velocity anomaly, ${\bar{q}}_{850}$ is the climatological 850-hPa specific humidity, and g is the gravity. TH, DY, and NL indicate the thermodynamic contribution from changes in mean moisture, the dynamic contribution from changes in ISO circulation, and their nonlinear interaction, respectively.

### Wave-activity flux

The wave-activity flux (WAF) (*63*) was used to illustrate the propagation of intraseasonal Rossby wave packets. The formulation is given by

$$\begin{array}{c} W=\frac{p\text{cos}\phi }{2|U|}\\ \left(\begin{array}{c} \frac{U}{a^{2}\text{cos}\phi }\left[{\left(\frac{\partial \psi^{\prime \prime }}{\partial \lambda }\right)}^{2}-\psi^{\prime \prime }\frac{\partial^{2}\psi^{\prime \prime }}{\partial \lambda^{2}}\right]+\frac{V}{a^{2}\text{cos}\phi }\left[\frac{\partial \psi^{\prime \prime }}{\partial \lambda }\frac{\partial \psi^{\prime \prime }}{\partial \phi }-\psi^{\prime \prime }\frac{\partial^{2}\psi^{\prime \prime }}{\partial \lambda \partial \phi }\right]\\ \frac{U}{a^{2}\text{cos}\phi }\left[\frac{\partial \psi^{\prime \prime }}{\partial \lambda }\frac{\partial \psi^{\prime \prime }}{\partial \phi }-\psi^{\prime \prime }\frac{\partial^{2}\psi^{\prime \prime }}{\partial \lambda \partial \phi }\right]+\frac{V}{a^{2}}\left[{\left(\frac{\partial \psi^{\prime \prime }}{\partial \phi }\right)}^{2}-\psi^{\prime \prime }\frac{\partial^{2}\psi^{\prime \prime }}{\partial \phi^{2}}\right] \end{array}\right) \end{array}$$ (6)

where **W** is the horizontal WAF vector; ϕ and λ represent the latitude and longitude, respectively; a is Earth’s radius (=$6.37\times 10^{6}$ m); and *p* is the normalized pressure (pressure/1000 hPa). The term U=(U,V) denotes the climatological seasonal mean basic flow, while $\psi^{\prime \prime }$ is the perturbation stream function. **W** represents the WAF associated with intraseasonal (10- to 90-day) variability.

### Analyses of future changes in the ISO using CMIP6 simulations

Because of biases in the simulation of the tropical ISO in CMIP6 models, *K*-means clustering was ineffective. Instead, we examined the propagation characteristics using lead-lag regression analyses of 10- to 90-day precipitation anomalies regressed onto the base point over the equatorial Indian Ocean (65°E to 80°E, 5°S to 5°N). Precipitation anomalies were first standardized using each model’s historical climatology, and the resulting regression patterns were then rescaled by the model-specific SD of 10- to 90-day precipitation to restore the physical amplitude of the associated rainfall anomalies. Considering data availability, we define 1986 to 2000 as the earlier epoch (E1) and 2001 to 2014 as the recent epoch (E2) (fig. S6D).

For the analysis of mid-latitude ISO variability, simulated 250-hPa meridional wind anomalies were projected onto the second EOF mode of observed 200-hPa meridional wind anomalies, which resembles the mid-latitude ISO circulation pattern associated with NWSA occurrences, to derive the PC time series of the mid-latitude ISO variability (fig. S9E). Mid-latitude ISO events were defined as cases in which PC values exceeded 1 SD for at least three consecutive days. Because of limitations in daily data availability or significant simulation biases, only 17 of 27 models (shown in fig. S15D) were used to analyze mid-latitude ISO variability.

### Sensitivity experiments using a coupled general circulation model

To assess the influence of mid-latitude ISOs on NWSA flood events, we conducted sensitivity experiments with the Geophysical Fluid Dynamics Laboratory LOAR version of CM2.5. LOAR uses a 2°-by-2° atmospheric grid with 32 vertical levels and a 1°-by-1° ocean grid. The control experiment was integrated for 200 years under constant 1990 radiative forcing to provide a stable baseline free from anthropogenic trends. In the sensitivity experiment, the model’s prognostic variables within 15°S to 15°N were nudged toward their 90-day low-pass–filtered components from the control run (the LP90 experiment), thereby suppressing tropical subseasonal variability with periods shorter than 90 days while retaining lower-frequency signals. Comparison between the control and LP90 simulations enables isolation of the mid-latitude ISO influence. Because long integrations of LP90 occasionally exhibited reduced stability, we used years 11 to 50 for analysis; extending the analysis period did not change the conclusions.
