# Supplementary material for: Rising flood risks in semiarid South Asia driven by changing intraseasonal oscillations under global warming
Source: Sci Adv. 2026 Apr 3;12(14):eaea0082. doi: 10.1126/sciadv.aea0082 (PMC13048259; doi:10.1126/sciadv.aea0082)
Supplement: Supplementary file 1 — Figs. S1 to S17 Table S1 [file sciadv.aea0082_sm.pdf]

Science Advances
AAAS

Supplementary Materials for

Rising flood risks in semiarid South Asia driven by changing intraseasonal oscillations under global warming

Jinhui Xie *et al.*

Corresponding author: Pang-Chi Hsu, pangchi@nuist.edu.cn

Sci. Adv. **12**, eaea0082 (2026)
DOI: 10.1126/sciadv.aea0082

This PDF file includes:

Figs. S1 to S17
Table S1

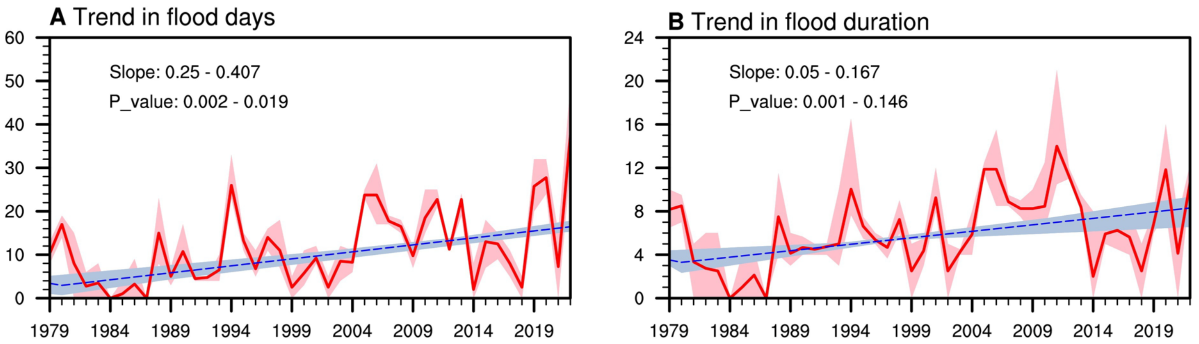

Supplementary Fig. 1. Time series of Northwestern South Asia (NWSA) flood days and flood duration. Time series of (**A**) NWSA flood days (units: days) and (**B**) flood duration (units: days). Red lines represent the ensemble mean from four datasets (CPC, ERA5, MERRA-2, and MSWEP), with shading indicating the inter-dataset range. Blue dashed lines denote the corresponding linear trends, with trend slopes and associated *p*-values shown in the top left corner. Trends were estimated using the Sen-slope method and their significance was tested with the Mann-Kendall test.

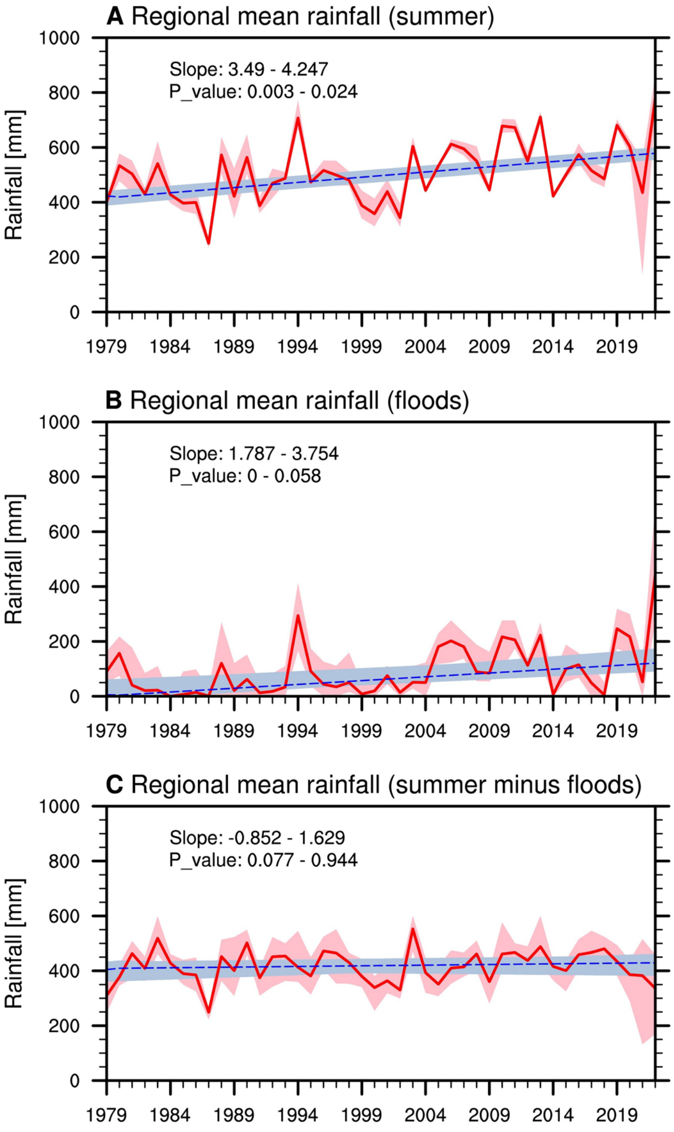

Supplementary Fig. 2. Sensitivity testing for the selection of Weighted Average of Precipitation (WAP) index parameters. Similar to Fig. 1D, but showing results using different *N* values (ranging from 0 to 44, Eq. 1) for the WAP index, based on four rainfall datasets (CPC, ERA5, MERRA-2, and MSWEP).

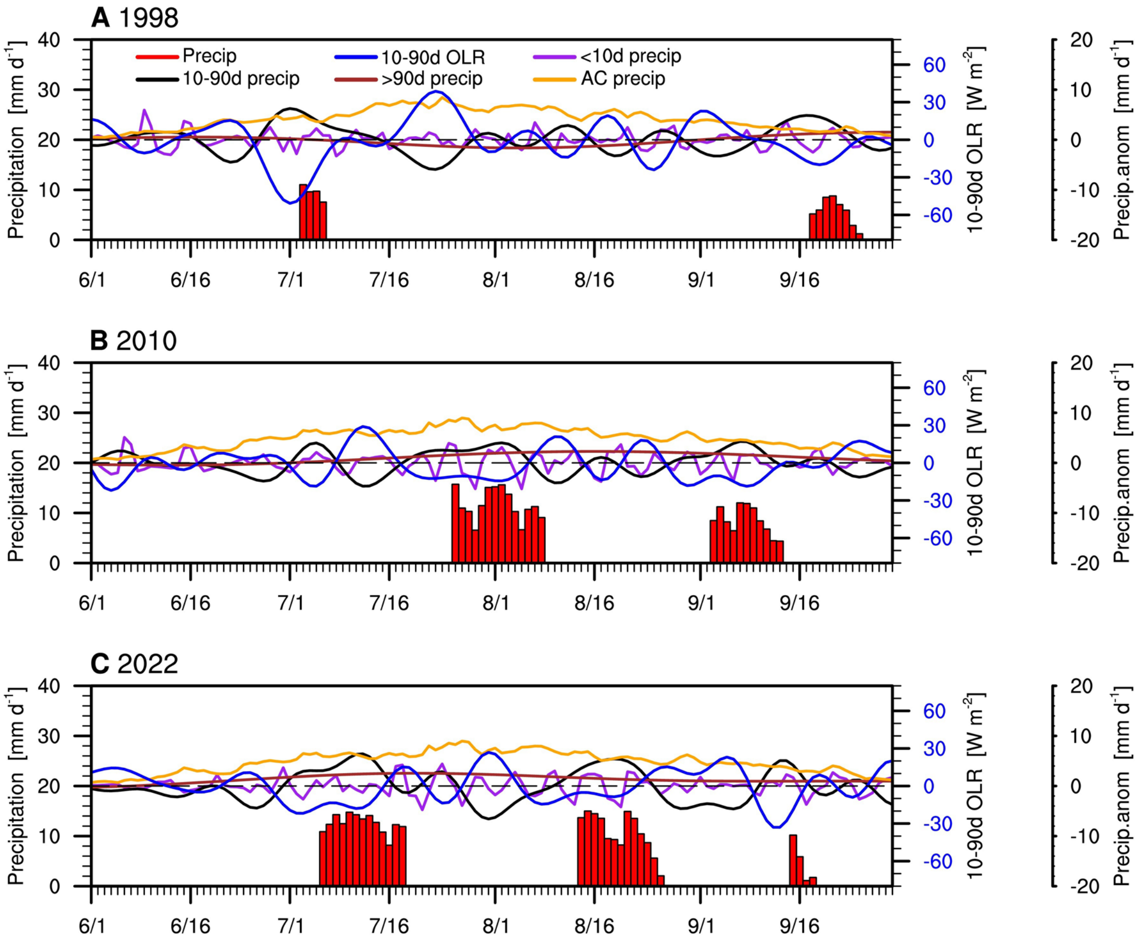

Supplementary Fig. 3. Rainfall and outgoing longwave radiation (OLR) anomalies associated with flooding events. Rainfall associated with flooding events (red bars; units: mm day^{-1}; left *y*-axis) in the (**A**) 1998, (**B**) 2010, and (**C**) 2022 summers, along with the evolution of 10–90-day OLR anomalies averaged over NWSA (blue line; units: W m^{-2}; right blue *y*-axis). Negative OLR anomalies indicate enhanced convection. The different timescale rainfall components (units: mm day^{-1}; right black *y*-axis) are displayed by the curves of various colors in the upper part of this panel: synoptic component (<10-day; purple curves); 10–90-day (black curves); >90-day (brown curves); and annual cycle (AC; orange curves). Rainfall data and OLR data were derived from ERA5 and NOAA, respectively.

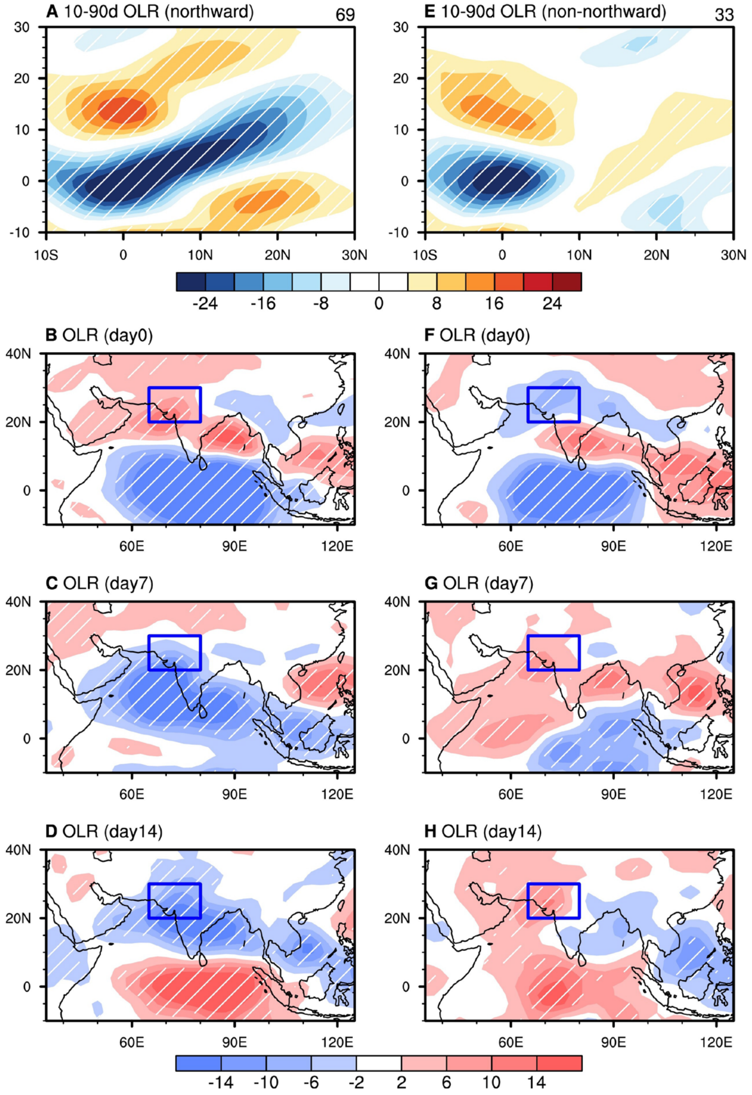

Supplementary Fig. 4. Spatiotemporal characteristics of OLR anomalies during different types of Intraseasonal Oscillation (ISO) events. (**A**) Latitude–time diagram of 10–90-day OLR anomalies (shading; units: W m^{-2}) composited from northward-propagating ISO events along 65°E–80°E. The number of events is indicated in the top right corner. The white dashed lines indicate regions where the anomalies are statistically significant at the 95% confidence level. (**B**–**D**) Composites of 10–90-day OLR anomalies (shading; units: W m^{-2}) at 0, 7, and 14 days after the tropical ISO convective anomaly reaches its maximum over the equatorial Indian Ocean (65°E–80°E, 5°S–5°N). The blue box delineates NWSA. (**E**–**H**) Same as (A–D) but for non-northward-propagating ISO events.

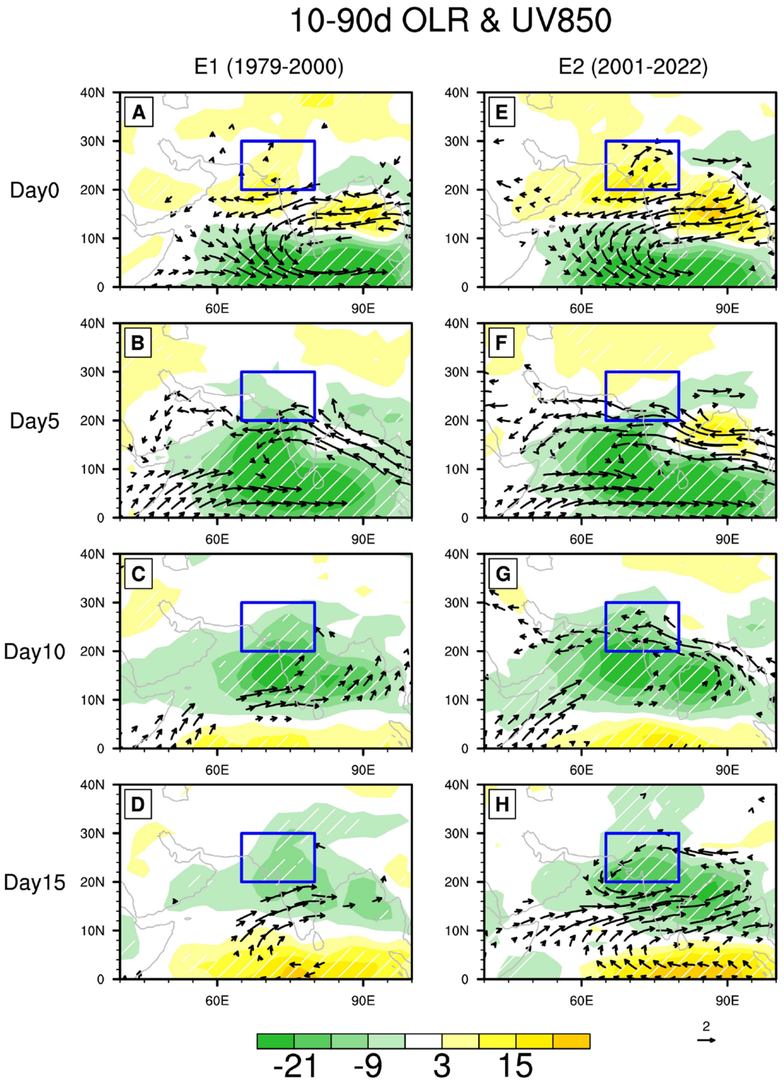

Supplementary Fig. 5. Composites of 10–90-day OLR and 850-hPa wind anomalies associated with tropical ISO events during two epochs. (**A**–**D**) Composites of 10–90-day OLR anomalies (shading; units: W m^{-2}) and 850-hPa wind anomalies (vectors; units: m s^{-1}; only statistically significant anomalies at the 95% confidence level are displayed) during the E1 period (1979–2000), shown at 0, 5, 10, and 15 days after tropical ISO events initiate over the equatorial Indian Ocean (65°E–80°E, 5°S–5°N). (**E**–**H**) Same as (A–D) but for the E2 period (2001–2022). Wind field data and OLR data were derived from ERA5 and NOAA, respectively. White hatched areas indicate regions where anomalies are statistically significant at the 95% confidence level. Blue boxes delineate NWSA.

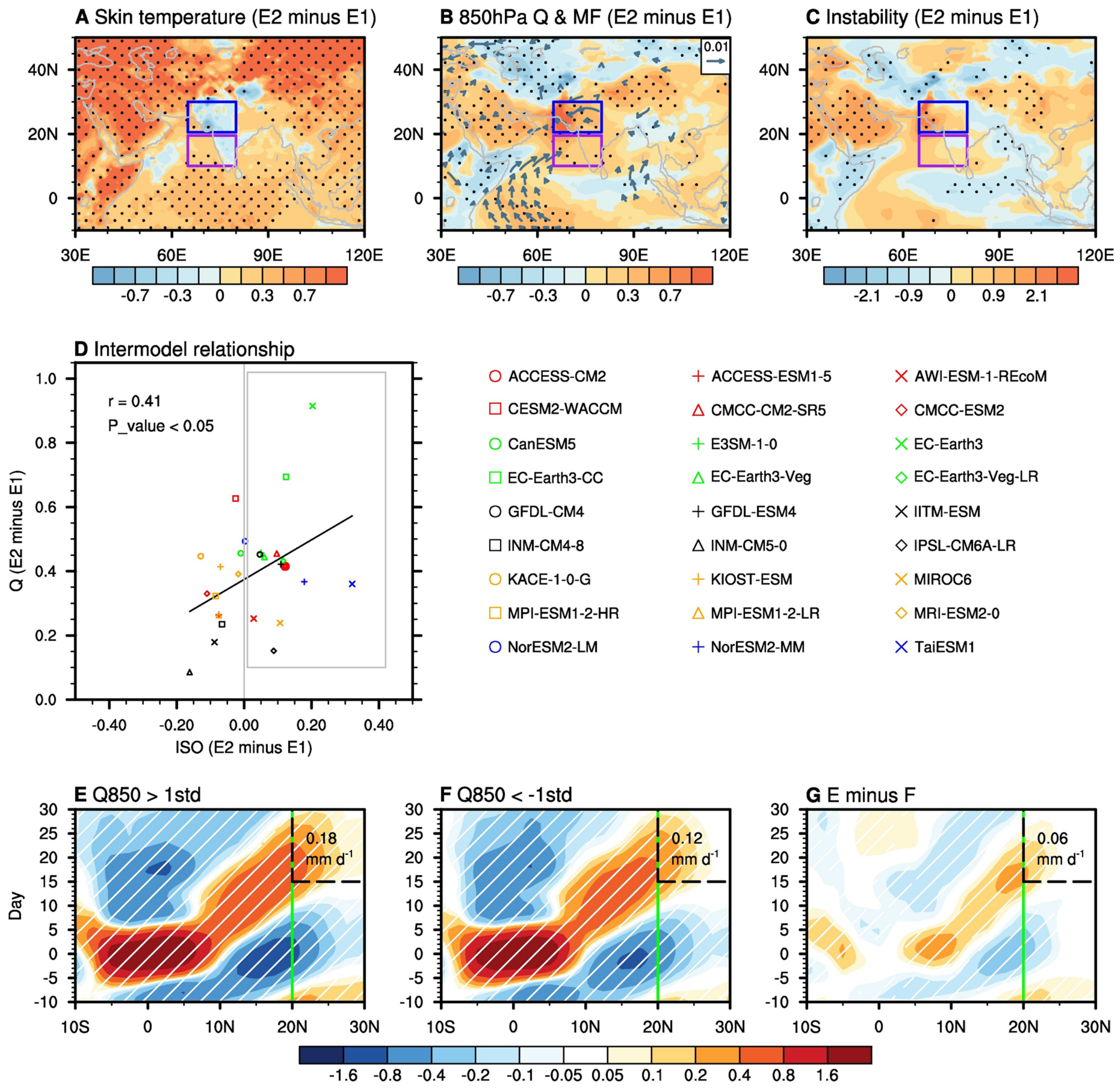

Supplementary Fig. 6. The role of the background conditions in tropical ISO propagation. Differences in the summer-mean (June–September, JJAS) atmospheric variables between the recent and earlier epochs (2001–2022 minus 1979–2000): (**A**) Skin temperature (units: K), (**B**) 850-hPa specific humidity (Q; shading; units: 10^{-3} kg kg^{-1}) and moisture flux (MF; vectors; units: kg m kg^{-1} s^{-1}), and (**C**) convective instability index (units: K), defined as the difference in equivalent potential temperature between the lower (925–700 hPa) and middle (400–300 hPa) levels, where positive values indicate a convectively unstable atmosphere. Source: ERA5. Dotted areas in (A–C) indicate regions where differences are statistically significant at the 95% confidence level. Blue boxes delineate NWSA, while purple boxes denote the northern Arabian Sea. (**D**)

Scatter plot of the 850-hPa humidity change over the northern Arabian Sea (2001–2014 minus 1987–2000; *y*-axis; units: 10^{-3} kg kg^{-1}) versus the tropical ISO-related rainfall change averaged over NWSA (units: mm day^{-1}) from CMIP6 historical simulations. The ISO-related rainfall over NWSA is derived from the NWSA-averaged values of 10−90-day precipitation fields regressed onto precipitation over the equatorial Indian Ocean (65°E–80°E, 5°S–5°N) at a lag of 15–30 days (i.e., the quantities within the black dashed boxes in the bottom panels). The black line represents the linear regression fit, with the correlation coefficient labeled in the top left corner. The gray box highlights 15 models that successfully simulate the observed strengthening of tropical ISO. (**E**) Latitude–time evolution of the tropical ISO-related rainfall (units: mm day^{-1}), represented by 10−90-day precipitation fields regressed onto precipitation over the equatorial Indian Ocean (65°E–80°E, 5°S–5°N), along 65°E–80°E longitude, from piControl experiments of the 15 models during summers when the 850-hPa moisture over the purple box exceeds one standard deviation. (**F**–**G**) Same as (E) but for summers with decreased moisture (below –1 standard deviation) and their differences (E minus F). White hatched areas in (E–G) indicate regions where at least 66% of the models agree on the sign of the changes. Green vertical lines mark the southern boundary latitude of NWSA.

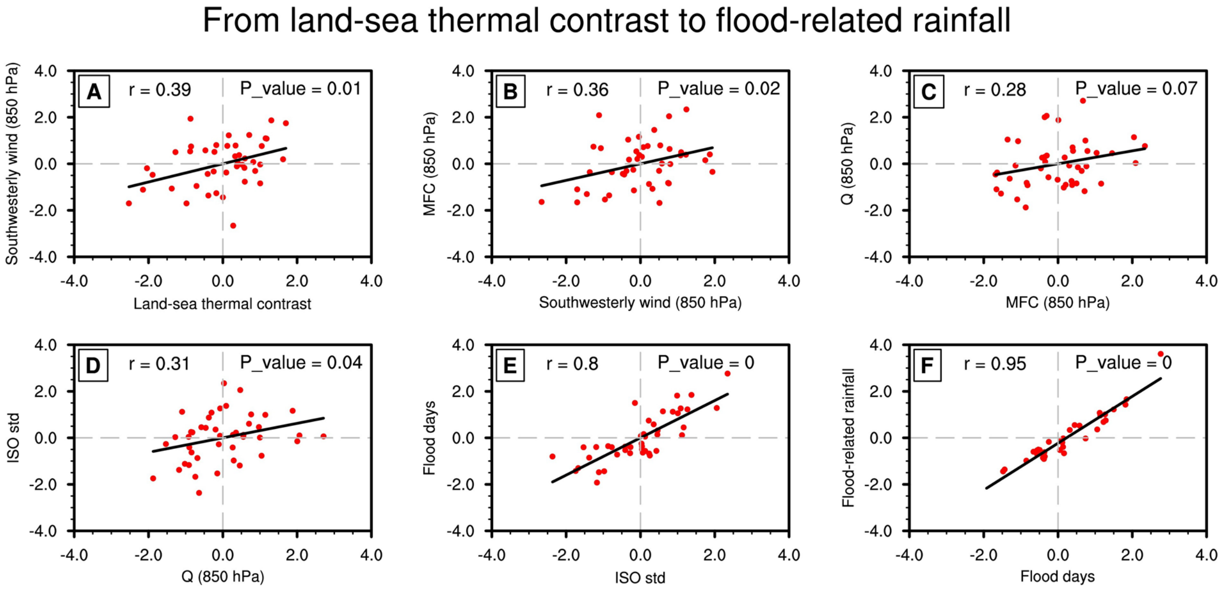

Supplementary Fig. 7. Scatter relationship between background conditions and flood occurrence. (**A**) Land–sea temperature difference (land: 40°E–80°E, 20°N–40°N; ocean: 40°E–80°E, 10°S–20°N) versus 850-hPa wind speed over the Arabian Sea (10°N–30°N, 40°E–80°E). (**B**) 850-hPa wind speed over the Arabian Sea versus 850-hPa moisture flux convergence over the northern Arabian Sea and NWSA (65°E–80°E, 10°N–30°N). (**C**) Moisture flux convergence versus local specific humidity over the northern Arabian Sea and NWSA. (**D**) Local specific humidity versus intraseasonal rainfall variability, measured by 10–90-day rainfall standard deviation, over NWSA. (**E**) Intraseasonal rainfall variability versus flood frequency. (**F**) Flood frequency versus flood-related rainfall, based on summer averages during 1979–2022. Each red dot represents the yearly mean, and black lines show the linear regression fit. Correlation coefficients and *p*-values (calculated using the *t*-test) are indicated in the upper portion of each panel. All metrics are normalized prior to plotting.

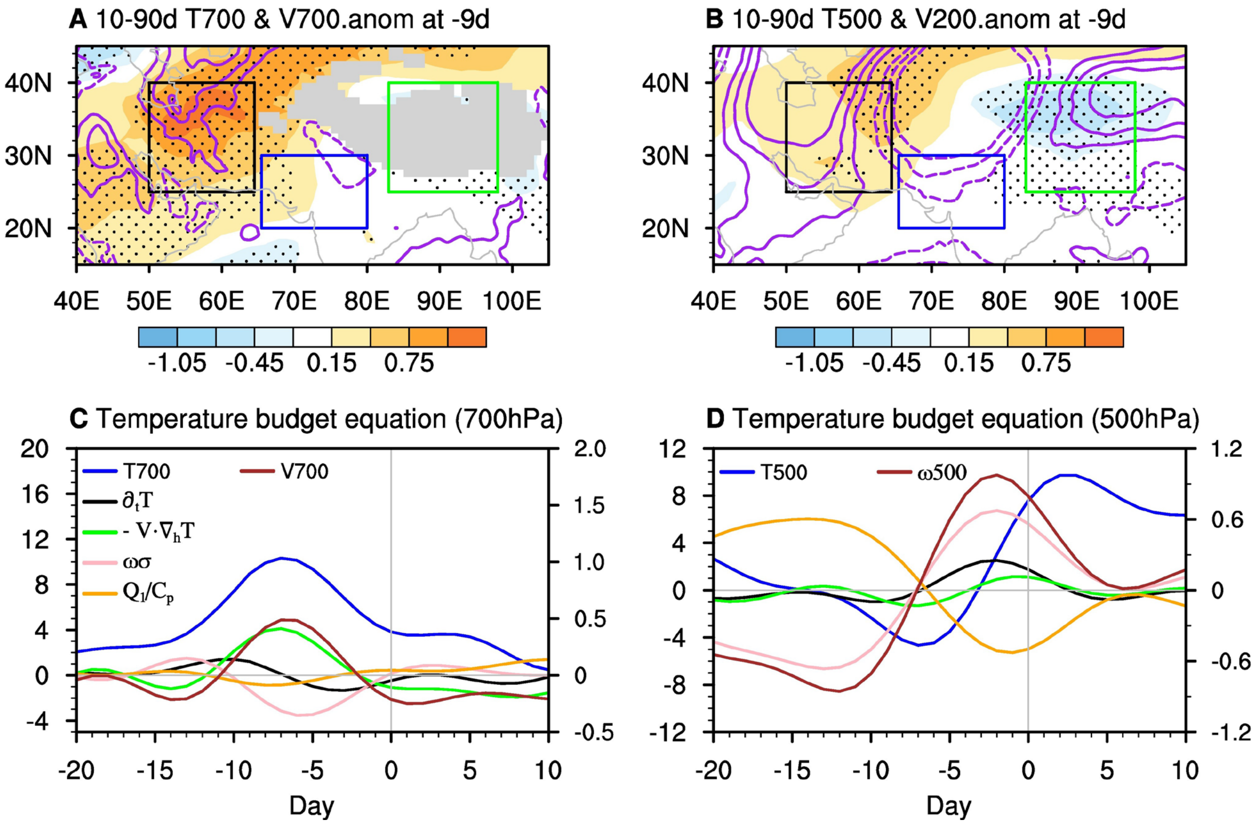

Supplementary Fig. 8. Thermodynamic anomalies associated with the midlatitude ISO preceding NWSA floods. (**A**) Composites of 700-hPa temperature anomalies (T700; shading; units: K) and meridional wind anomalies (V700; contours with an interval of 0.4 from −1.2 to 1.2; units: m s^{-1}) at 9 days before NWSA flood events. Stippling indicates anomalies significant at the 90% confidence level. The blue box denotes NWSA, the black and green boxes mark the regions used to diagnose surface-temperature anomalies over the Iranian Plateau–Arabian Peninsula and Tibetan Plateau, respectively. (**B**) Same as (A), but for 500-hPa temperature and 200-hPa meridional wind anomalies. (**C**) Evolution of V700 (brown line; units: m s^{-1}; right *y*-axis), temperature budget terms at 700-hPa (units: 10^{-5} K s^{-1}; left *y*-axis) over the Iranian Plateau–Arabian Peninsula based on ERA5. Brown, blue, black, green, pink, and orange lines denote the variations of V700, T, $\partial T / \partial t$, $-\mathbf{V} \cdot \nabla T$, ωσ, Q_1/C_p, respectively. The *x*-axis indicates days before (negative), during (0; gray vertical line), and after (positive) flood onset. (**D**) Similar to (C), but showing the 500-hPa temperature budget over the Tibetan Plateau and the corresponding 500-hPa vertical-motion anomalies (brown; units: 10^{-2} Pa s^{-1}).

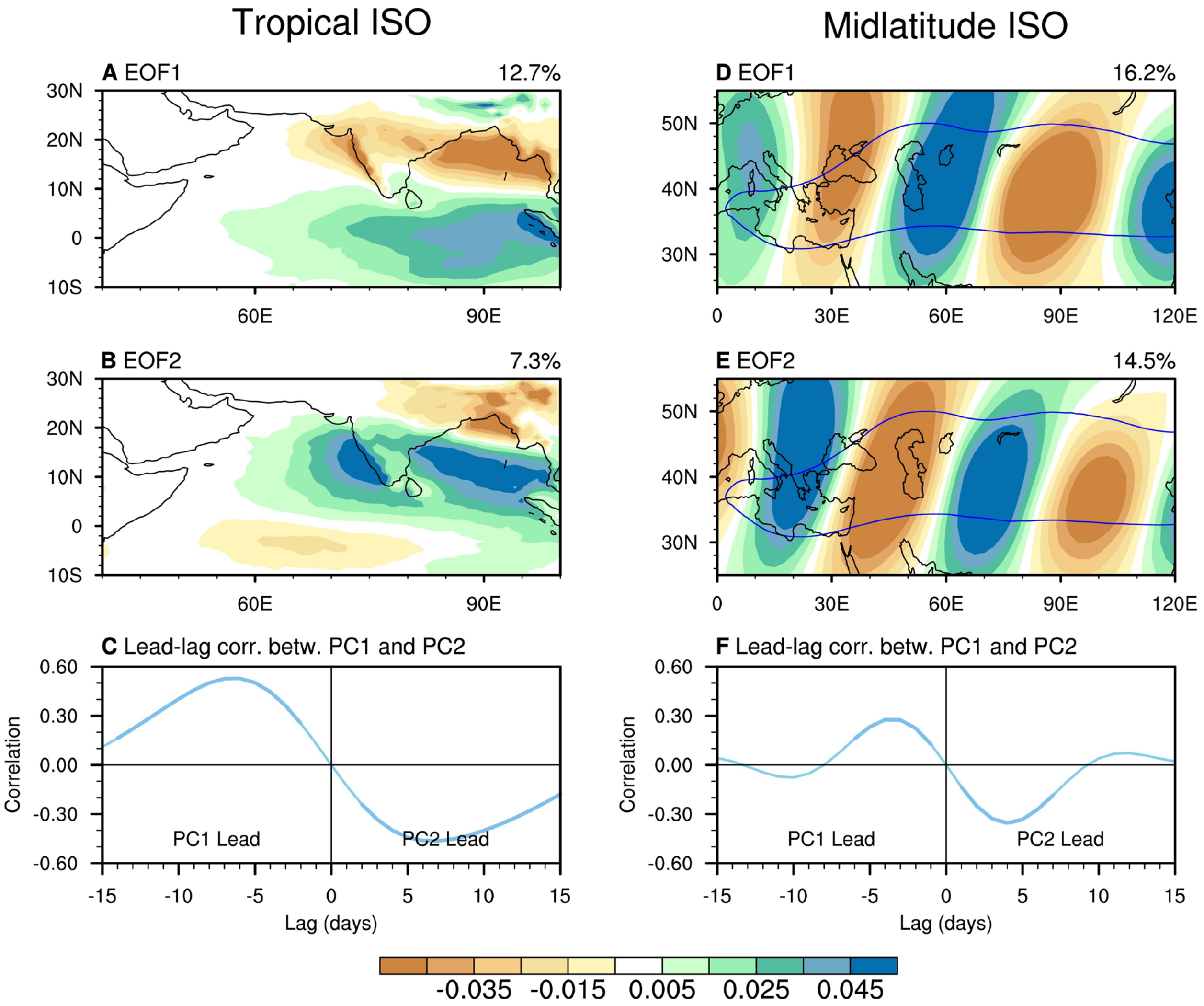

Supplementary Fig. 9. Definition of tropical and midlatitude ISO modes from Empirical Orthogonal Function (EOF) analysis. (**A**–**B**) Spatial distributions of the first (EOF1) and second (EOF2) EOF modes of the May–October (MJJASO) 10–90-day rainfall anomalies, representing key patterns of tropical ISO variability. (**C**) Lead–lag correlation between the principal components (PCs) of EOF1 and EOF2, with the dark blue section of the line indicating statistically significant values at the 95% confidence level. (**D**–**F**) Same as (A–C) but for the EOF analysis of MJJASO 10–90-day 200-hPa meridional wind anomalies over the Eurasian westerly jet region (0°–120°E, 25°N–55°N). The blue contour in (D–E) represents the MJJASO-averaged 200-hPa zonal wind (U200) of 18 m s^{-1}, marking the westerly jet location.

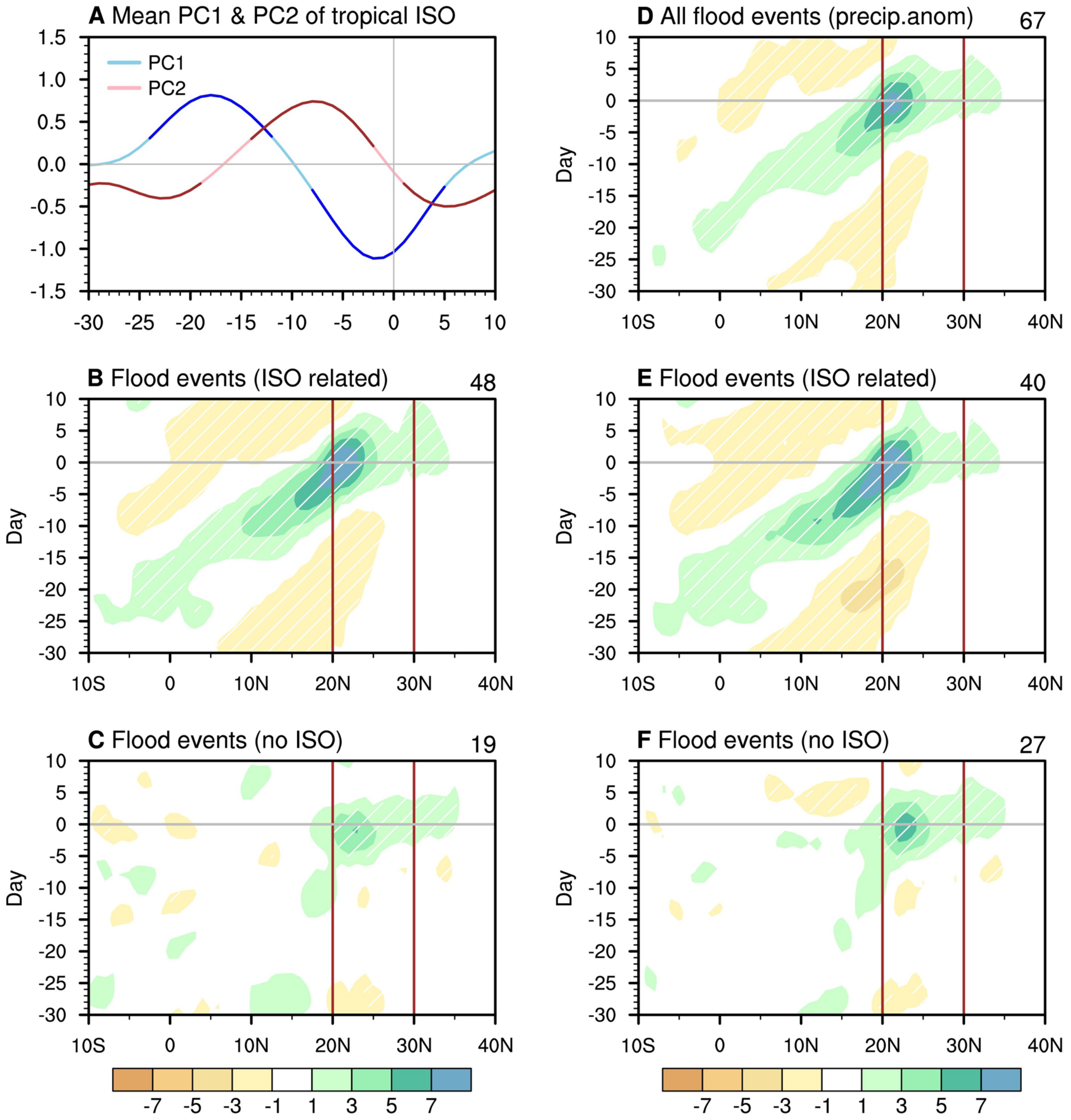

Supplementary Fig. 10. Latitude–time evolution of 10–90-day rainfall anomalies for tropical ISO-related flood events over NWSA. (**A**) Temporal evolution of the tropical ISO modes PC1 (blue line) and PC2 (red line) before and after flood events, with the dark section of the line indicating statistically significant values at the 95% confidence level. The *x*-axis indicates days before (negative), during (0; gray vertical line), and after (positive) flood onset. (**B**) Latitude–time diagram of 10–90-day rainfall anomalies (shading; units: mm day^{-1}), averaged over 65°E–80°E, composited for flood events associated with the tropical ISO. Negative and positive values on the *y*-axis indicate days before and after flood onset. (**C**) is similar to (B), but shows the composites

for flood events unrelated to the tropical ISO. White hatched areas indicate regions where the results are statistically significant at the 95% confidence level. Brown vertical lines mark the boundary latitude of NWSA, and the gray horizontal line marks the onset of flood events. The numbers in the upper-right corner indicate the number of events used for the composite. (**D**–**F**) are similar to (B) but showing composites of all flood events, flood events associated with the tropical ISO as defined by the second method (Methods), and flood events not associated with the tropical ISO, respectively.

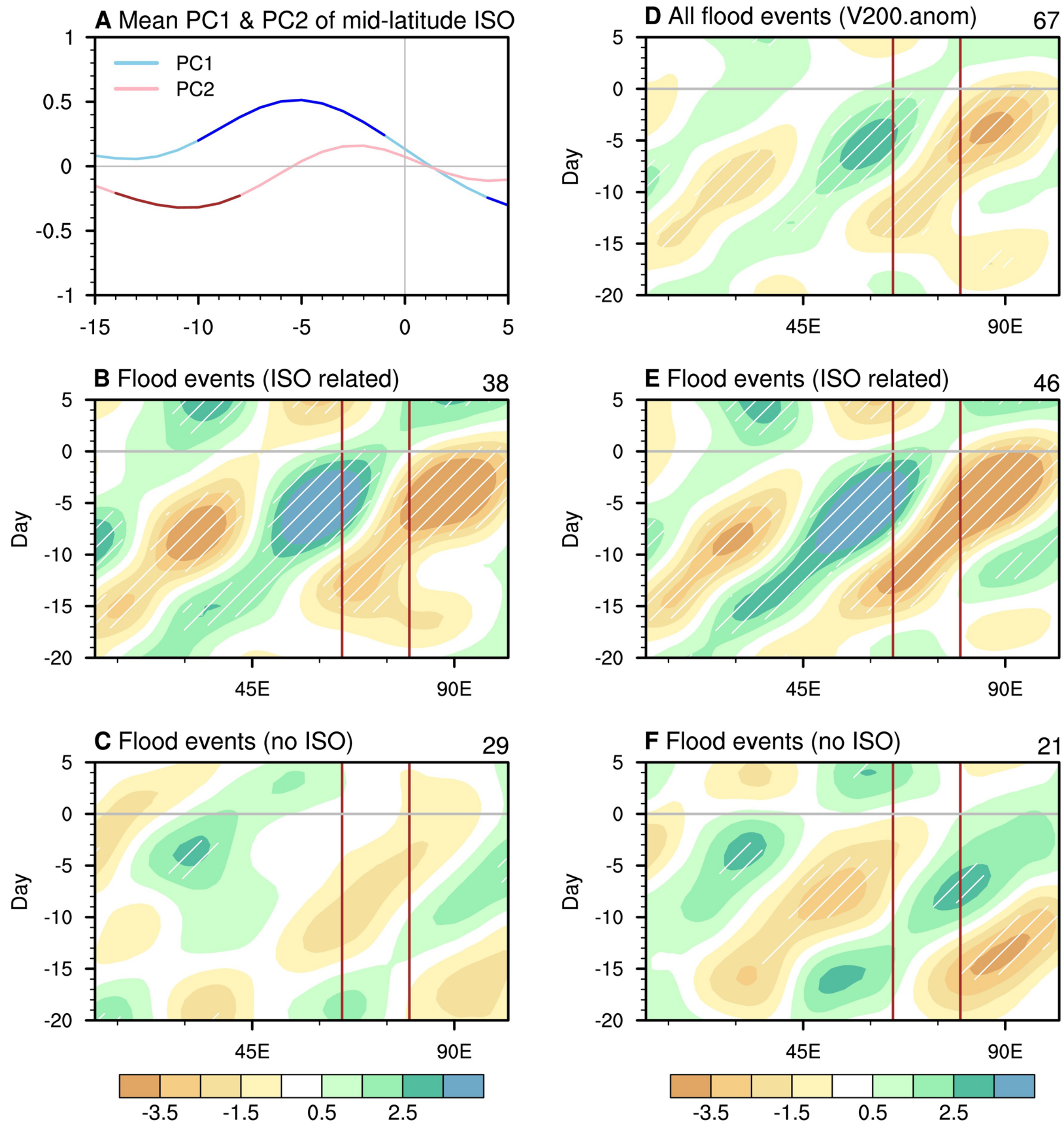

Supplementary Fig. 11. Longitude–time evolution of 10–90-day V200 anomalies for midlatitude ISO-related flood events over NWSA. Similar to Supplementary Fig. 10, but showing composites of midlatitude ISO-related flood events.

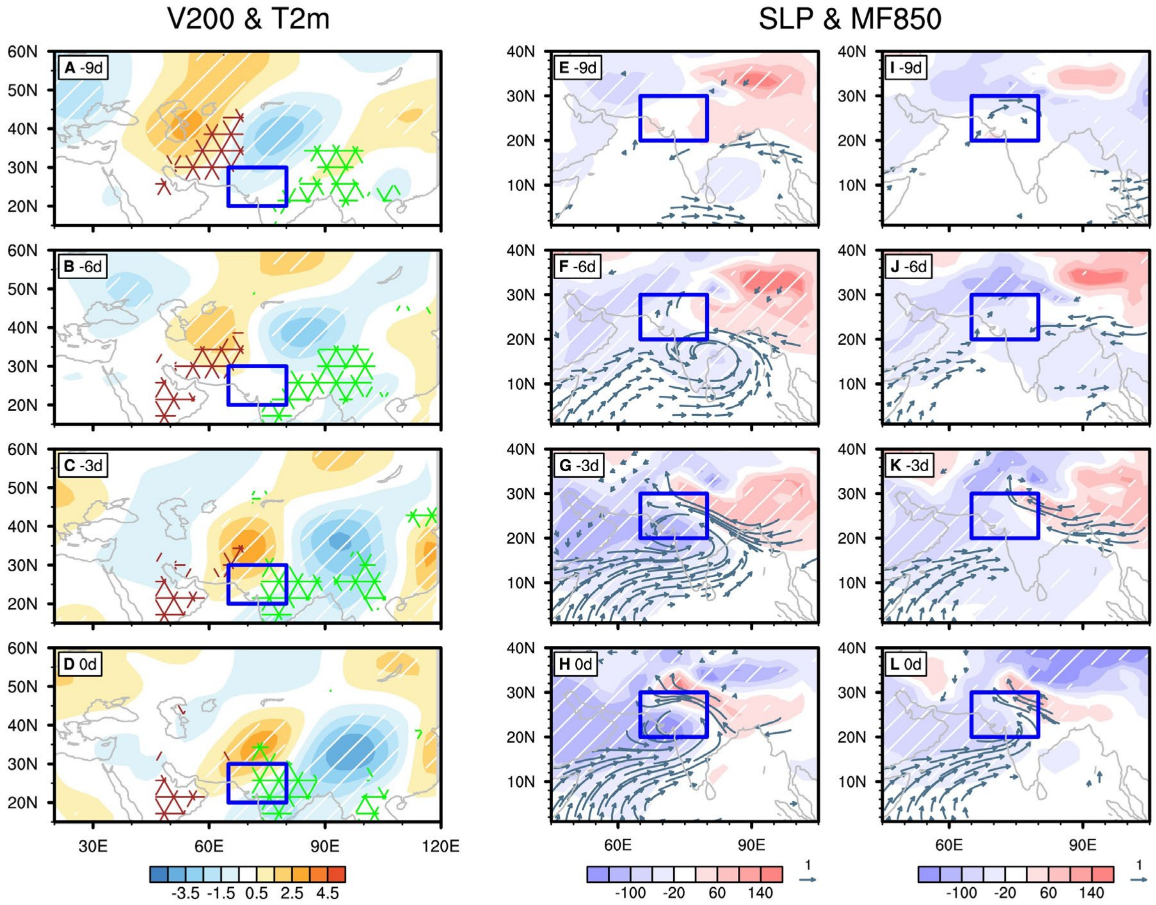

Supplementary Fig. 12. Sensitivity experiments verifying the influence of the midlatitude ISO on NWSA flood events. Similar to Fig. 3, but for the GFDL sensitivity experiments. Panels (**A**–**H**) show the composites based on the NWSA flood events identified in the control simulation, while panels (**I**–**L**) show the corresponding composites from the LP90 experiment.

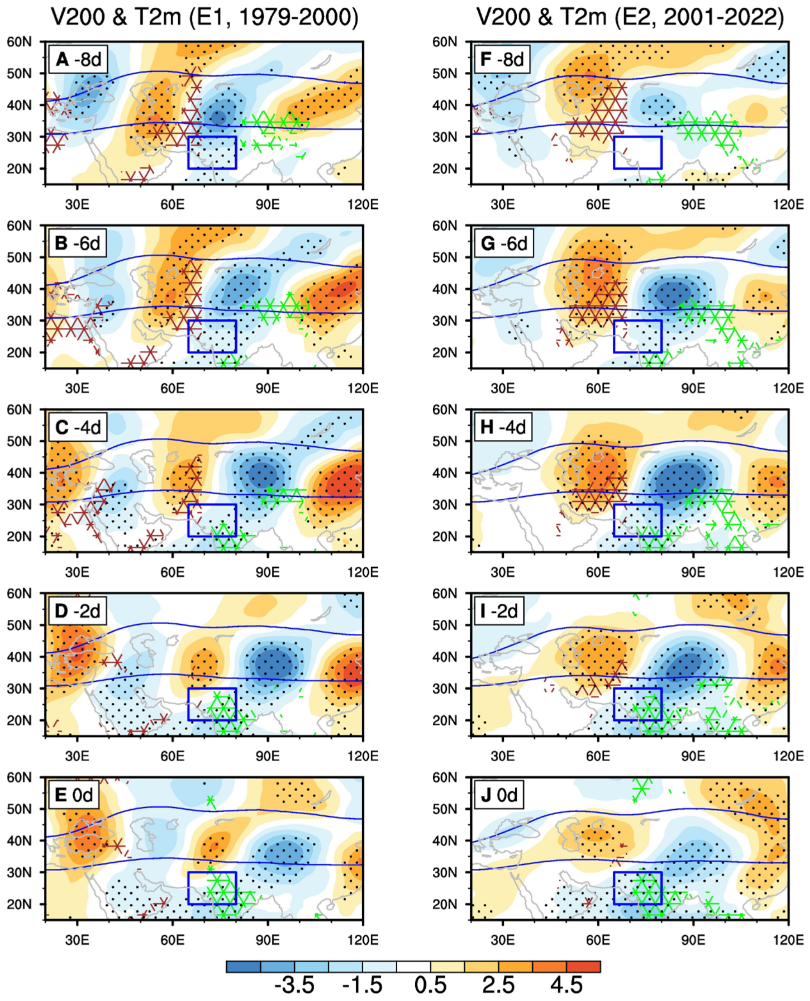

Supplementary Fig. 13. Composites of 200-hPa meridional wind and 2-meter air temperature anomalies around NWSA flood events during two epochs. (**A**–**E**) Composites of 10–90-day 200-hPa meridional wind anomalies (V200; shading; units: m s^{-1}) and 2-meter air temperature anomalies (T2m; red crosses denote positive anomalies, and green crosses denote negative anomalies; units: K; only anomalies significant at the 90% confidence level are shown) at 8, 6, 4, 2, and 0 days before NWSA flood events, respectively, during the E1 period (1979–2000). The blue curve represents the JJAS-averaged U200 contour of 18 m s^{-1}, indicating the location of the westerly jet. (**F**–**J**) Same as (A–E) but for the E2 period (2001–2022). Blue boxes delineate NWSA. Dotted areas denote regions significant at the 90% confidence level based on a *t*-test.

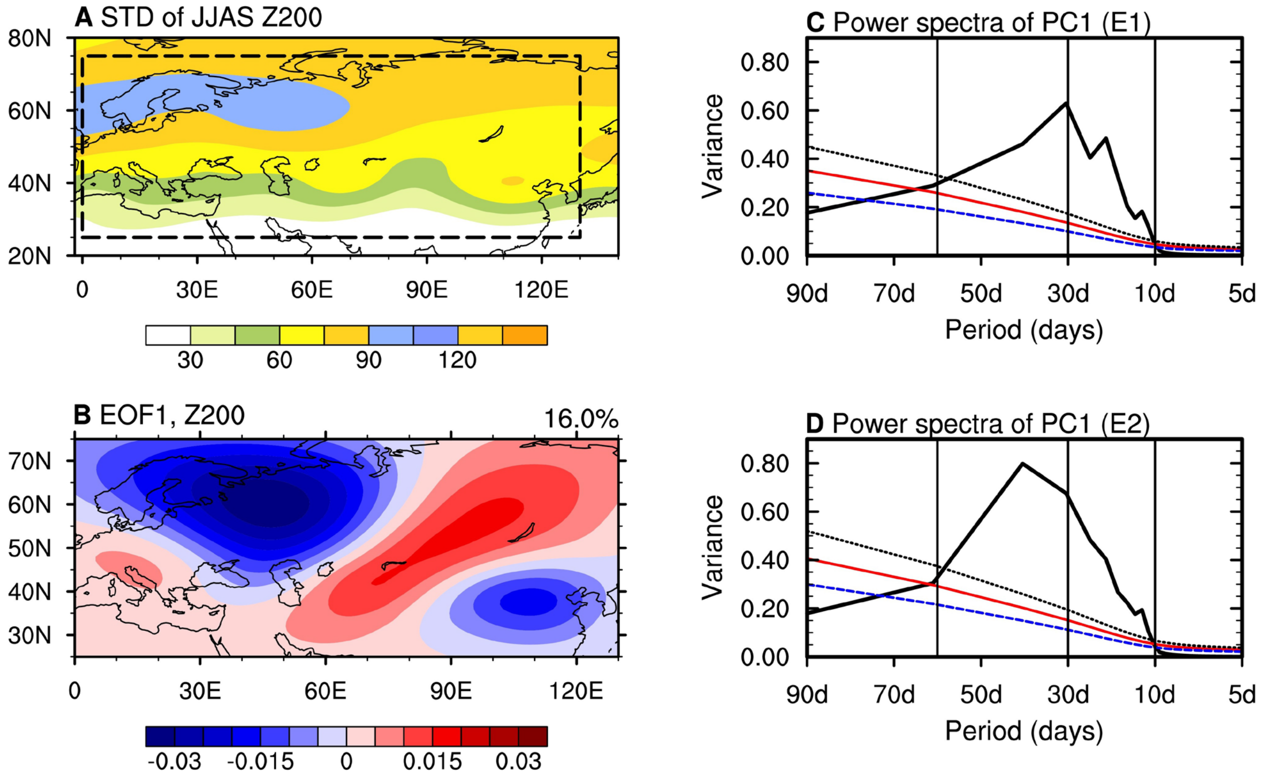

Supplementary Fig. 14. Variability and EOF analysis of JJAS 200-hPa geopotential height anomalies over Eurasia. (**A**) Standard deviations of 10–90-day 200-hPa geopotential height anomalies (Z200; shading; units: gpm) during JJAS, highlighting regions of active Z200 variability. (**B**) First EOF mode of JJAS Z200 anomalies over the Eurasian region outlined by the black dotted box in (A). The explained variance is indicated in the upper right corner. (**C**–**D**) Power spectra of the corresponding PC1 during E1 (1979–2000) and E2 (2001–2022), respectively. The red solid line represents the Markov red noise spectrum, while the blue and black dashed lines indicate the *a priori* and *a posteriori* 90% confidence levels, respectively.

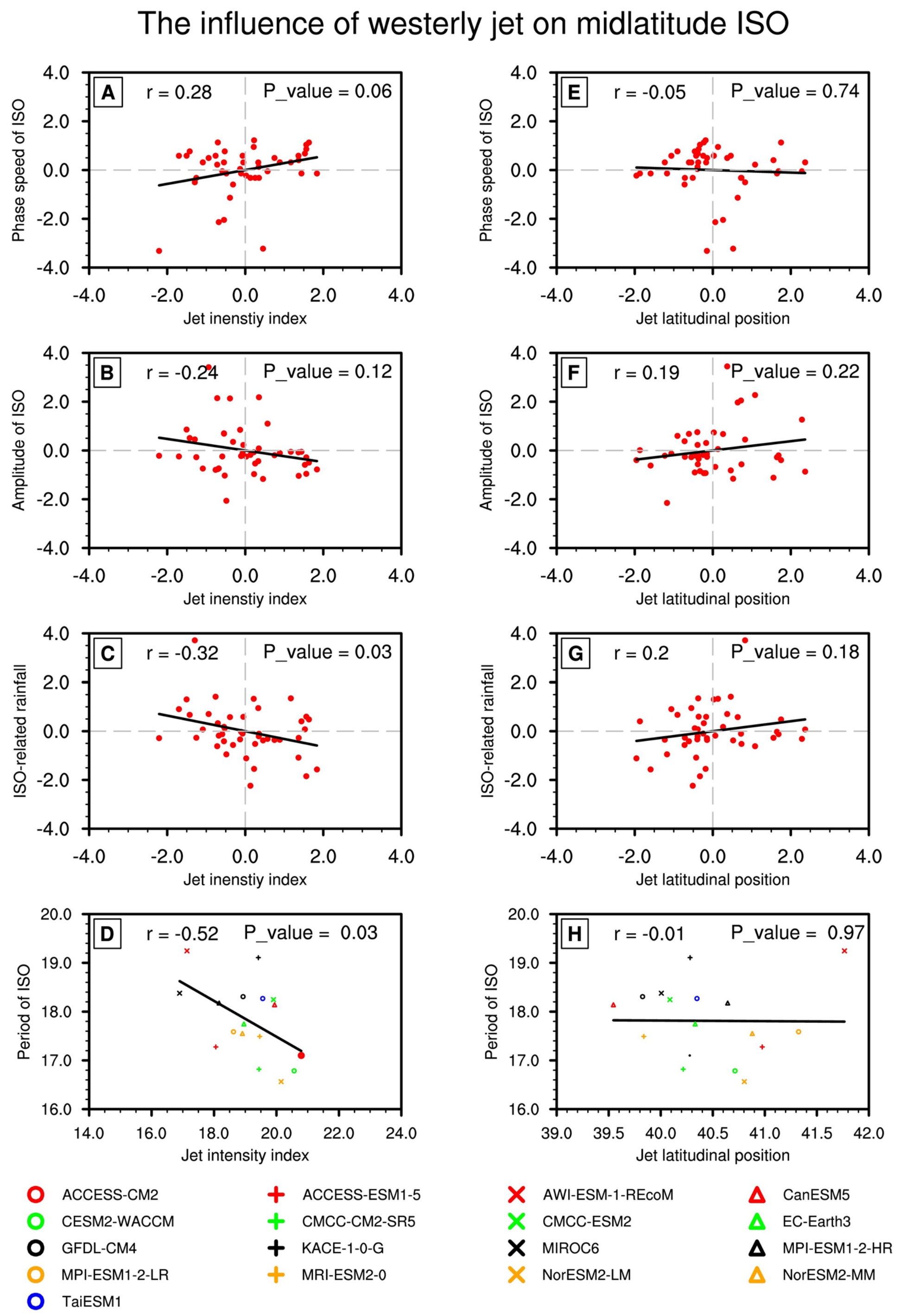

Supplementary Fig. 15. Impact of the westerly jet stream on midlatitude ISO activity. (A–C) Relationships between the westerly jet intensity index—defined as the area-mean 200-hPa zonal wind over the core jet region (25°E–125°E, 30°N–50°N)—and key midlatitude ISO metrics. Midlatitude ISO events are identified as periods when PC1 > 0.5 standard deviation (SD) for at

least 5 days and PC2 undergoes a negative-to-positive transition (Methods). (A) ISO phase speed, (B) ISO amplitude (mean value of the first principal component time series over the 7-day window centered on the peak of midlatitude ISO events), (C) ISO-related rainfall over NWSA (mean rainfall within 10 days following the peak of midlatitude ISO events) during 1979–2022. Each red dot denotes the annual mean value, and the black line indicates the linear regression fit. Correlation coefficients and *p*-values (calculated using a *t*-test) are given in the upper portion of each panel. All variables are normalized prior to plotting. (**D**) Scatter plot of the westerly jet intensity index versus the dominant periodicity (peak in the power spectra) of the second EOF mode of V200 (Methods), which represents the midlatitude ISO mode here, in CMIP6 historical simulations. The black solid line represents the linear regression fit, with the correlation coefficient displayed in the upper corner. (**E**–**H**) Same as (A–D), but showing relationships with the westerly jet latitudinal-position index, defined as the wind-speed-weighted mean latitude of the maximum zonal wind across each longitude band within the jet core region (25°E–125°E, 30°N–50°N).

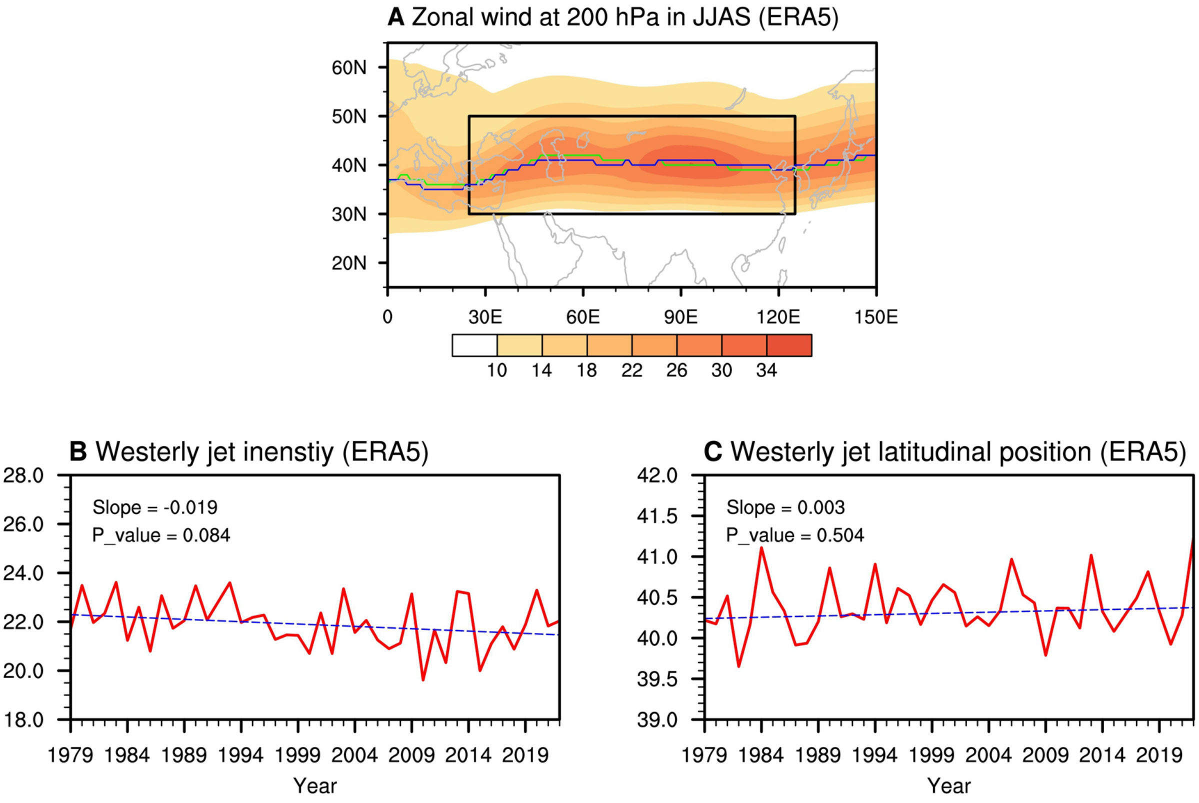

Supplementary Fig. 16. Long-term changes in the Eurasian westerly jet. (**A**) Climatological 200-hPa zonal wind (U200; shading; units: m s^{-1}) over Eurasia from ERA5 reanalysis. The black box marks the core region of the westerly jet. Green and blue lines indicate the jet axis, defined by the maximum U200, for E1 (1979–2000) and E2 (2001–2022), respectively. (**B**) Time series of the westerly jet index (red line; units: m s^{-1}). The linear trend is shown by the blue dashed line (units: m s^{-1} $year^{-1}$), with the trend slope and *p*-value given in the top-left corner. (**C**) Similar to (B) but for the westerly jet latitudinal-position index.

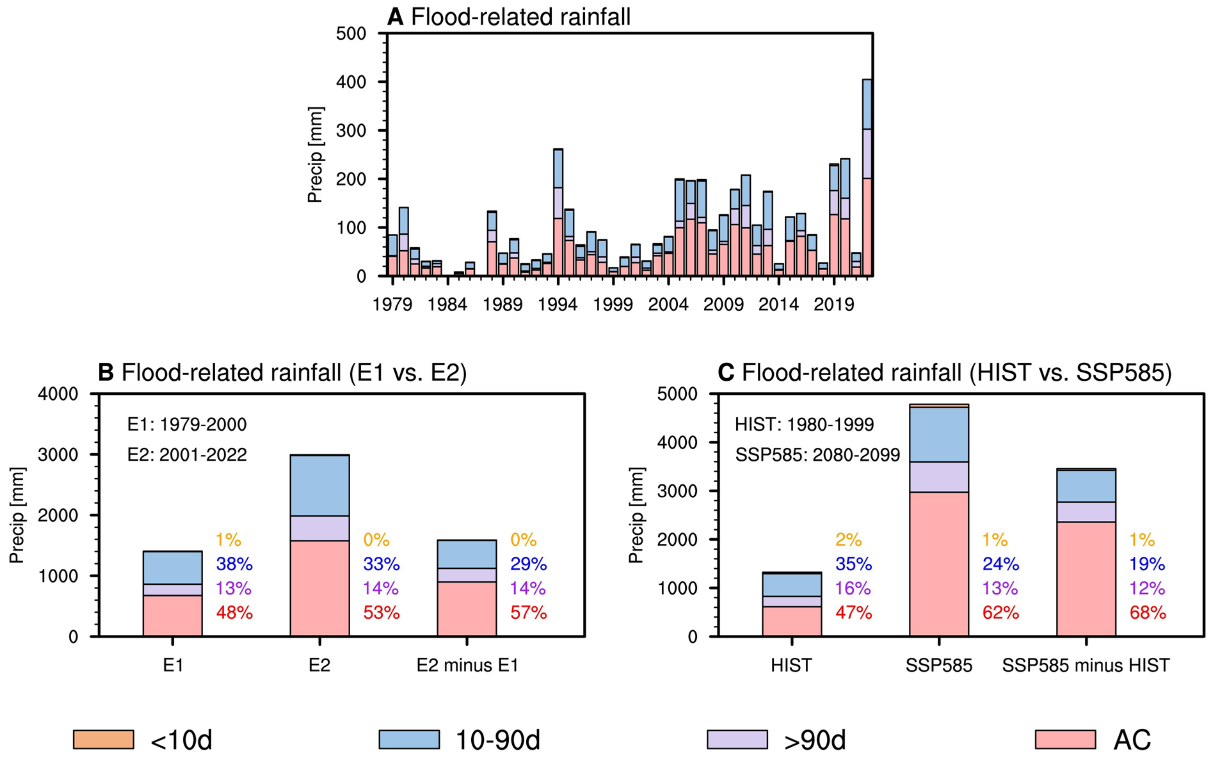

Supplementary Fig. 17. Relative contributions of different timescale components to flood-related rainfall. (**A**) Contributions of different timescale components to flood-related rainfall from 1979 to 2022, based on the ensemble mean of CPC, ERA5, MERRA-2, and MSWEP. Rainfall components (units: mm) are displayed by colored bars: synoptic (<10-day; orange), 10–90-day (light blue), >90-day (purple), and annual cycle (AC; pink) components. (**B**) Relative contributions of each timescale component to flood-related rainfall during E1 (1979–2000), E2 (2001–2022), and their differences (E2 minus E1). Values to the right of each bar indicate the fractional contribution of that component. (**C**) Same as (B), but for CMIP6 historical (HIST) simulations (1980–1999), SSP5-8.5 projections (2080–2099), and their differences.

Supplementary Table S1 Details of the 27 CMIP6 models used in this study.

Model acronym	Institution/Country	Variables used	Atmosphere resolution Lat × Lon
ACCESS-CM2	CSIRO-ARCCSS, Australia	u, v, q, pr	144 × 192
ACCESS-ESM1-5	CSIRO-ARCCSS, Australia	u, v, q, pr	145 × 192
AWI-ESM-1-REcoM	AWI, Germany	u, v, q, pr	96 × 192
CanESM5	CCCMA, Canada	u, v, q, pr	64 × 128
CESM2-WACCM	NCAR, United States	u, v, q, pr	192 × 288
CMCC-CM2-SR5	CMCC, Italy	u, v, q, pr	192 × 288
CMCC-ESM2	CMCC, Italy	u, v, q, pr	192 × 288
E3SM-1-0	E3SM, United States	u, v, q, pr	180 × 360
EC-Earth3	EC-Earth-Consortium, Europe	u, v, q, pr	256 × 512
EC-Earth3-CC	EC-Earth-Consortium, Europe	u, v, q, pr	256 × 512
EC-Earth3-Veg	EC-Earth-Consortium, Europe	u, v, q, pr	256 × 512
EC-Earth3-Veg-LR	EC-Earth-Consortium, Europe	u, v, q, pr	160 × 320
GFDL-CM4	NOAA GFDL, United States	u, v, q, pr	90 × 144
GFDL-ESM4	NOAA GFDL, United States	u, v, q, pr	180 × 288
IITM-ESM	IITM, India	u, v, q, pr	94 × 192
INM-CM4-8	INM, Russia	u, v, q, pr	120 × 180
INM-CM5-0	INM, Russia	u, v, q, pr	120 × 180
IPSL-CM6A-LR	IPSL, France	u, v, q, pr	143 × 144
KACE-1-0-G	NIMS-KMA, Korea	u, v, q, pr	144 × 192
KIOST-ESM	KIOST, Korea	u, v, q, pr	96 × 192
MIROC6	MIROC, Japan	u, v, q, pr	128 × 256
MPI-ESM1-2-HR	MPI-M, Germany	u, v, q, pr	192 × 384
MPI-ESM1-2-LR	MPI-M, Germany	u, v, q, pr	96 × 192
MRI-ESM2-0	MRI, Japan	u, v, q, pr	160 × 320
NorESM2-LM	NCC, Norway	u, v, q, pr	96 × 144
NorESM2-MM	NCC, Norway	u, v, q, pr	192 × 288
TaiESM1	TaiESM, Taiwan/China	u, v, q, pr	192 × 288
